# Pericentrin deficiency in smooth muscle cells augments atherosclerosis through HSF1-driven cholesterol biosynthesis and PERK activation

**DOI:** 10.1172/jci.insight.173247

**Published:** 2023-11-08

**Authors:** Suravi Majumder, Abhijnan Chattopadhyay, Jamie M. Wright, Pujun Guan, L. Maximilian Buja, Callie S. Kwartler, Dianna M. Milewicz

**Affiliations:** 1 Division of Medical Genetics, Department of Internal Medicine, McGovern Medical School, and; 2 Department of Pathology and Laboratory Medicine, The University of Texas Health Science Center at Houston, Houston, Texas, USA.

**Keywords:** Genetics, Vascular Biology, Atherosclerosis, Cell stress, Cholesterol

## Abstract

Microcephalic osteodysplastic primordial dwarfism type II (MOPDII) is caused by biallelic loss-of-function variants in pericentrin (*PCNT*), and premature coronary artery disease (CAD) is a complication of the syndrome. Histopathology of coronary arteries from patients with MOPDII who died of CAD in their 20s showed extensive atherosclerosis. Hyperlipidemic mice with smooth muscle cell–specific (SMC-specific) *Pcnt* deficiency (*Pcnt^SMC–/–^*) exhibited significantly greater atherosclerotic plaque burden compared with similarly treated littermate controls despite similar serum lipid levels. Loss of PCNT in SMCs induced activation of heat shock factor 1 (HSF1) and consequently upregulated the expression and activity of HMG-CoA reductase (HMGCR), the rate-limiting enzyme in cholesterol biosynthesis. The increased cholesterol biosynthesis in *Pcnt^SMC–/–^* SMCs augmented PERK signaling and phenotypic modulation compared with control SMCs. Treatment with the HMGCR inhibitor, pravastatin, blocked the augmented SMC modulation and reduced plaque burden in hyperlipidemic *Pcnt^SMC–/–^* mice to that of control mice. These data support the notion that *Pcnt* deficiency activates cellular stress to increase SMC modulation and plaque burden, and targeting this pathway with statins in patients with MOPDII has the potential to reduce CAD in these individuals. The molecular mechanism uncovered further emphasizes SMC cytosolic stress and HSF1 activation as a pathway driving atherosclerotic plaque formation independently of cholesterol levels.

## Graphical abstract

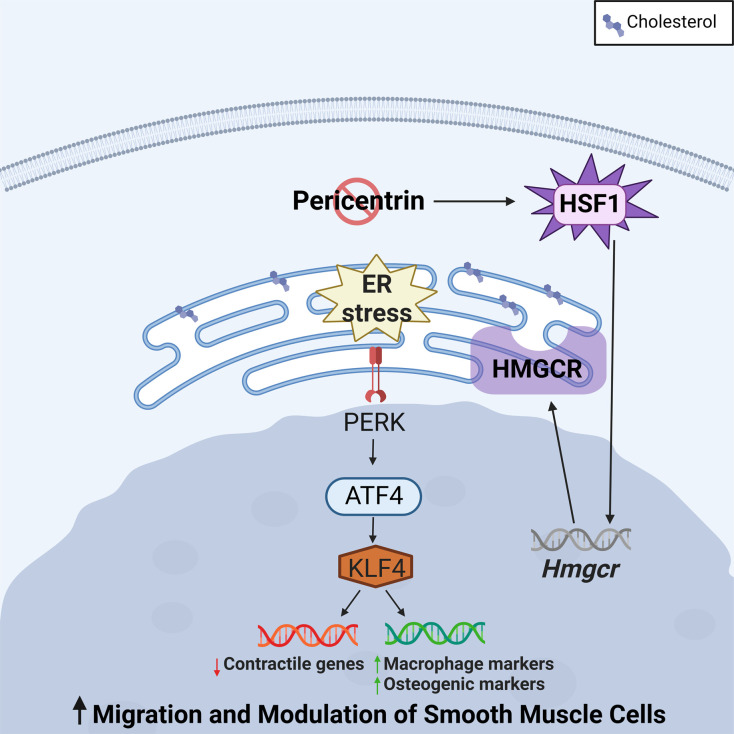

## Introduction

Atherosclerosis predisposes individuals to coronary artery disease (CAD), a leading cause of death globally. Lineage tracing and single-cell transcriptomic studies (scRNA-seq) have demonstrated that vascular smooth muscle cells (SMCs) undergo complex phenotypic modulation during atherosclerotic plaque formation in mice, characterized by downregulation of SMC differentiation genes (e.g., *Acta2*, *Myh11*), and variable increases in the expression of markers for stem cells (e.g., *Ly6a*), fibroblasts (*Fn1*, *Ecrg4*), macrophages (*Lgals3*), and chondrocyte-like cells (*Spp1*) (1–4). Exposing SMCs in culture to free cholesterol or oxidized low density lipoprotein (oxLDL) recapitulates many aspects of this phenotypic modulation due to the movement of cholesterol into the cell, triggering an unfolded protein response (UPR) in the endoplasmic reticulum (ER) and activating double-stranded RNA–activated protein kinase R (PKR)–like endoplasmic reticulum kinase (PERK). ATF4 levels increase with PERK signaling, which activates the transcription factor Krüppel-like factor 4 (KLF4) and drives phenotypic modulation of SMCs (5, 6). Hyperlipidemic mice with SMC-specific deficiency of either *Perk* (also designated *Eif2ak3*) or *Klf4* have a reduction in macrophage- and chondrocyte-like SMCs within plaques, and SMC-specific deletion of *Perk* in hyperlipidemic mice decreases atherosclerotic plaque by 80% when compared with similarly treated wild-type (WT) mice (1, 6, 7).

A pathogenic variant in *ACTA2*, p.R149C, predisposes individuals to both thoracic aortic disease and early-onset atherosclerosis in the absence of hyperlipidemia or other risk factors (8, 9). *Acta2^R149C/+^* mice do not develop thoracic aortic disease but have increased plaque formation on a hyperlipidemic background compared with similarly treated control mice, despite comparable lipid profiles (10, 11). The pathway linking a mutation in a contractile protein, the smooth muscle–specific isoform of α-actin (SMA, encoded by *ACTA2*), and increased atherosclerosis is initiated by misfolding and retention of the SMA R149C monomer in the cytoplasmic complex that folds all actins, the chaperonin-containing t-complex polypeptide 1 (CCT) (12). This retention leads to cytosolic stress and the activation of heat shock factor 1 (HSF1), which increases cellular cholesterol levels through increasing the expression of genes that encode enzymes involved in cholesterol biosynthesis, thus triggering ER stress, PERK signaling, and SMC modulation. These same pathways are also activated when cytosolic stress in SMCs is activated by heat shocking the cells (11). Treatment of the mice with a statin, pravastatin, that targets the rate-limiting enzyme of cholesterol biosynthesis, HMG-CoA reductase (HMGCR), decreases atherosclerotic burden in hyperlipidemic *Acta2^R149C/+^Apoe^–/–^* mice to levels similar to hyperlipidemic *Apoe^–/–^* mice, while lipid profiles remain the same (11, 13).

Microcephalic osteodysplastic primordial dwarfism type II (MOPDII) results from homozygous loss-of-function variants in *PCNT* (14). MOPDII patients have intrauterine growth retardation, severe but proportionate short stature, and pronounced microcephaly. Individuals with MOPDII also have early-onset and severe vascular diseases, including CAD, moyamoya disease (MMD), and intracranial aneurysms (15–17). *PCNT* encodes pericentrin, an integral component of the centrosome that serves as a multifunctional scaffold for anchoring protein complexes in cells. In particular, pericentrin is critical for the microtubule organizing center, which assembles the mitotic spindle during mitosis (18, 19). Defective cell division due to loss of pericentrin is hypothesized to be the etiology of the growth retardation, but the mechanisms driving the premature vascular diseases are unknown. Since HSF1 localizes to the kinetochore and spindle pole during mitosis, it could be also activated by the loss of pericentrin (20, 21). HSF1 also acts as a mitotic regulator, controlling chromosome segregation by elevating S326 phosphorylation and transcriptional activity of HSF1 during mitosis (22).

To identify the mechanism responsible for CAD in MOPDII, we initially assessed the coronary arteries in 2 MOPDII patients who died of CAD and confirmed their coronary arteries had histopathologic features of atherosclerosis. To test the hypothesis that loss of pericentrin in SMCs contributes to the atherosclerosis, hyperlipidemia was induced in SMC-specific *Pcnt*-deficient (*Pcnt^SMC–/–^*) and WT mice. *Pcnt^SMC–/–^* mice have increased atherosclerotic plaque burden compared with similarly treated WT mice, despite comparable lipid profiles. Using SMCs explanted from the aortas of *Pcnt^SMC–/–^* mice, we found that the same pathways that drive augmented phenotypic modulation and increased migration in *Acta2^R149C/+^* SMCs are activated in the *Pcnt^SMC–/–^* SMCs. Specifically, activation of HSF1 increases endogenous cholesterol biosynthesis, thus augmenting PERK signaling and SMC modulation. Furthermore, blocking cholesterol biosynthesis using pravastatin decreases atherosclerotic burden in *Pcnt^SMC–/–^* to levels similar to those of WT mice, once again without differences in serum lipid levels between these mice. Thus, these studies identify a mechanistic link between pericentrin loss in SMCs and augmented atherosclerotic burden in patients with MOPDII, and further emphasize the role of SMC cytosolic stress in driving atherosclerosis.

## Results

### Characterization of coronary artery atherosclerotic lesions in patients with MOPDII.

Coronary artery tissue was obtained at autopsy from 2 siblings of European descent with MOPDII. Patient 1, a 25-year-old male, had a past medical history of extracranial-intracranial bypass surgery for MMD, kidney transplantation for chronic kidney disease, and microsurgical clipping of a left vertebral artery aneurysm, along with hypertension and hypercholesterolemia, and was started on a statin at 20 years of age (17). He died of a myocardial infarct at the age of 25 years. Patient 2, a 30-year-old female, had a past medical history of stage IV chronic kidney disease, tracheomalacia status post tracheostomy, asthma, diastolic cardiac dysfunction, and CAD, and a statin was started when she was 22 years of age. During a paraesophageal hernia repair she suffered an acute ischemic stroke and developed a urinary tract infection, leading to acute kidney disease requiring dialysis. She subsequently suffered an acute myocardial infarction and sepsis, leading to her death. Both patients had loss-of-function variants in *PCNT* identified through diagnostic genetic testing.

**Figure 1:**
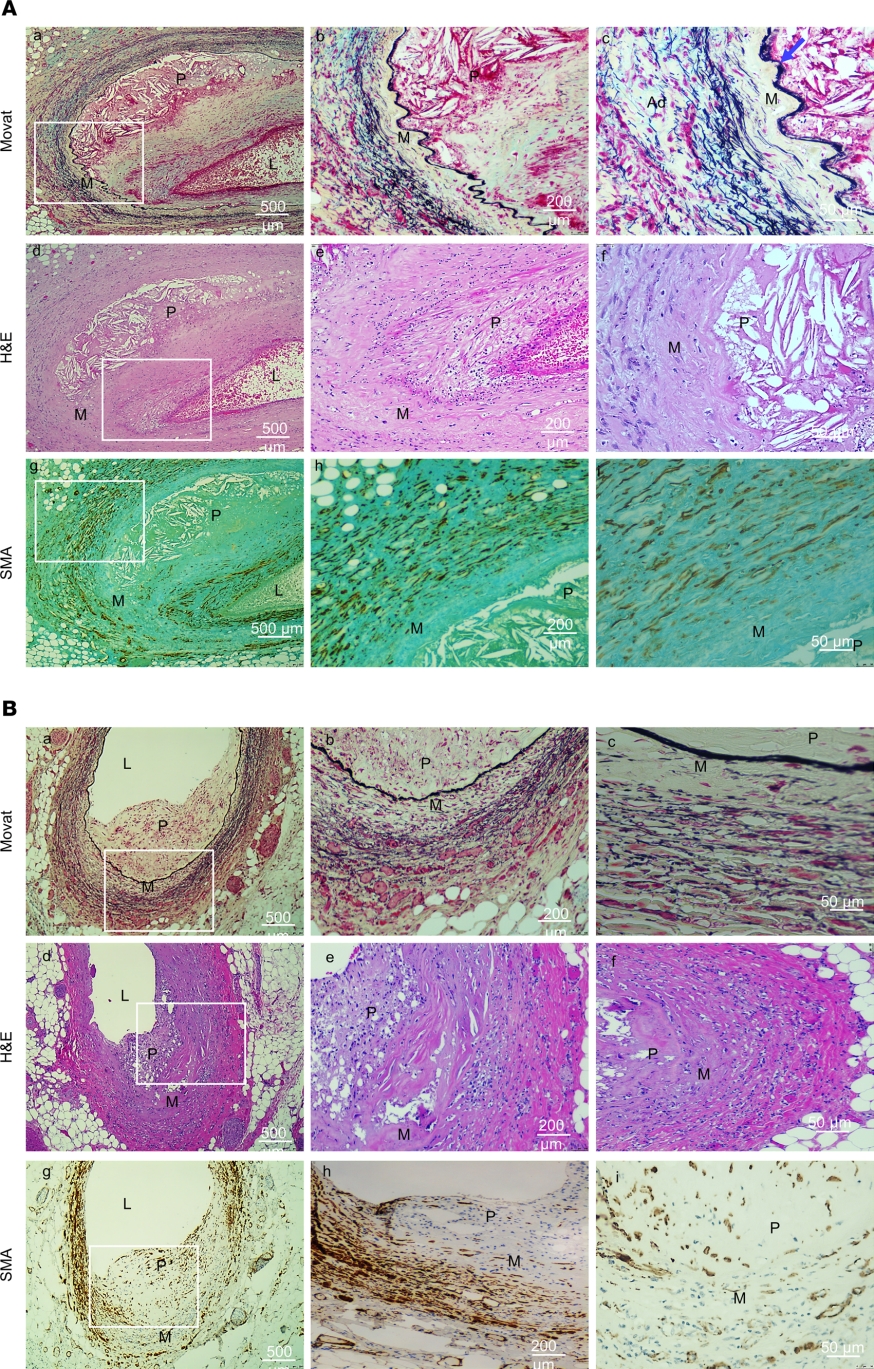
Characterization of coronary artery atherosclerotic lesions in patients with MOPDII. (**A**) Left anterior descending coronary artery from MOPDII Patient 1. Low-magnification image of Movat staining (a) reveals a large atherosclerotic plaque (P) in the left anterior descending coronary artery with a small lumen (L). Higher magnification image of the medial layer (M), shown in b and c, shows a well-formed internal elastic lamina (c, blue arrow), with an absence of cell staining in the medial layer and increased elastin deposition in the adventitial layer (Ad; the black elastin fibers are found in the adventitial layer). H&E staining (d–f) confirms loss of medial cells, along with cholesterol crystals in the atherosclerotic plaque. α-Smooth muscle actin (SMA) staining reveals large numbers of SMA-positive cells in the adventitia and fibrous cap (g and h) of the atherosclerotic plaque and an absence of SMA-positive cells in the medial layer (i). (**B**) Left main coronary artery bifurcation of MOPDII Patient 2. The low-magnification image of the Movat staining (a) reveals a large atherosclerotic plaque (P) with residual lumen (L). Higher-magnification images of the artery wall reveal an absence of cells in the medial layer (M) and elastosis (b and c). H&E staining (d–f) shows similar changes. SMA staining shows SMA-positive cells in a portion of the artery wall (g) and in the fibrous cap (h), with a paucity of SMA-positive cells in other areas of the medial wall (i). Scale bars: 500 μm (left), 200 μm (middle), and 50 μm (right).

At autopsy, Patient 1 had severe, stenotic coronary arteriopathy, with both the left and right coronary arteries showing variable degrees of intimal thickening and atheromatous changes (Figure 1A). The proximal to mid-right coronary artery had complete stenosis with superimposed thrombus. The proximal to mid-left anterior descending artery had marked stenosis, also with a superimposed thrombus. There were extensive areas of myocardial infarction, involving both the left and right ventricle and the interventricular septum, most prominent in the posterior and lateral walls of the left ventricle. At autopsy, Patient 2 was also found to have a stenotic coronary arteriopathy, with 90% occlusion of the right coronary artery, and 30% occlusion of the left main, left anterior descending and left circumflex coronary arteries (Figure 1B). There was evidence of multiple prior myocardial infarctions, involving the posterior-lateral left ventricle and posterior interventricular septum, with patchy interstitial fibrosis throughout the right ventricle, posterior interventricular septum, and posterior-lateral left ventricle with associated myocyte hypertrophy. The aortic arch had numerous calcifications and the descending aorta showed medial calcification and intimal hyperplasia.

Histologic examination of coronary arteries from both patients revealed large atheromatous plaques containing cells, calcification, and cholesterol crystals surrounded by macrophages, loss of SMCs in the medial layer, and increased accumulation of elastic fiber fragments in the adventitial layer (Figure 1, A and B). Immunostaining for SMA, a marker for SMCs, revealed SMA^+^ cells in the plaque and the adventitial layer, and very few vacuolated SMCs within the medial layer. Thus, these MOPDII plaques have features typical for atherosclerotic plaques, including cholesterol crystals, macrophage foam cells (stained for CD68, Supplemental Figure 1A; supplemental material available online with this article; https://doi.org/10.1172/jci.insight.173247DS1), and SMA^+^ cells in the plaques, but also have unique features, including prominent loss of SMCs in the medial layer and increased elastin deposition in the adventitial layer.

### SMC-specific deficiency of Pcnt in hypercholesterolemic mice increases atherosclerotic plaque burden.

To determine whether loss of *Pcnt* augments phenotypic modulation of SMCs and increases plaque burden, SMC-specific *Pcnt*-deficient mice were generated using a constitutive *SM22*α*-Cre* driver crossed with *Pcnt^fl/fl^* mice (designated *Pcnt^SMC–/–^*). The experimental mice were maintained in a mixed background of C57BL/6 and 129S4/SvJaeSor. Littermate mice lacking the *SM22*α*-Cre* gene but with the floxed allele were used as controls and designated WT. SMCs were explanted from the ascending aortas of WT and *Pcnt^SMC–/–^* mice, and loss of *Pcnt* was confirmed through quantitative real-time PCR (qRT-PCR) and immunoblot analyses of pericentrin (Supplemental Figure 1, B and C).

**Figure 2:**
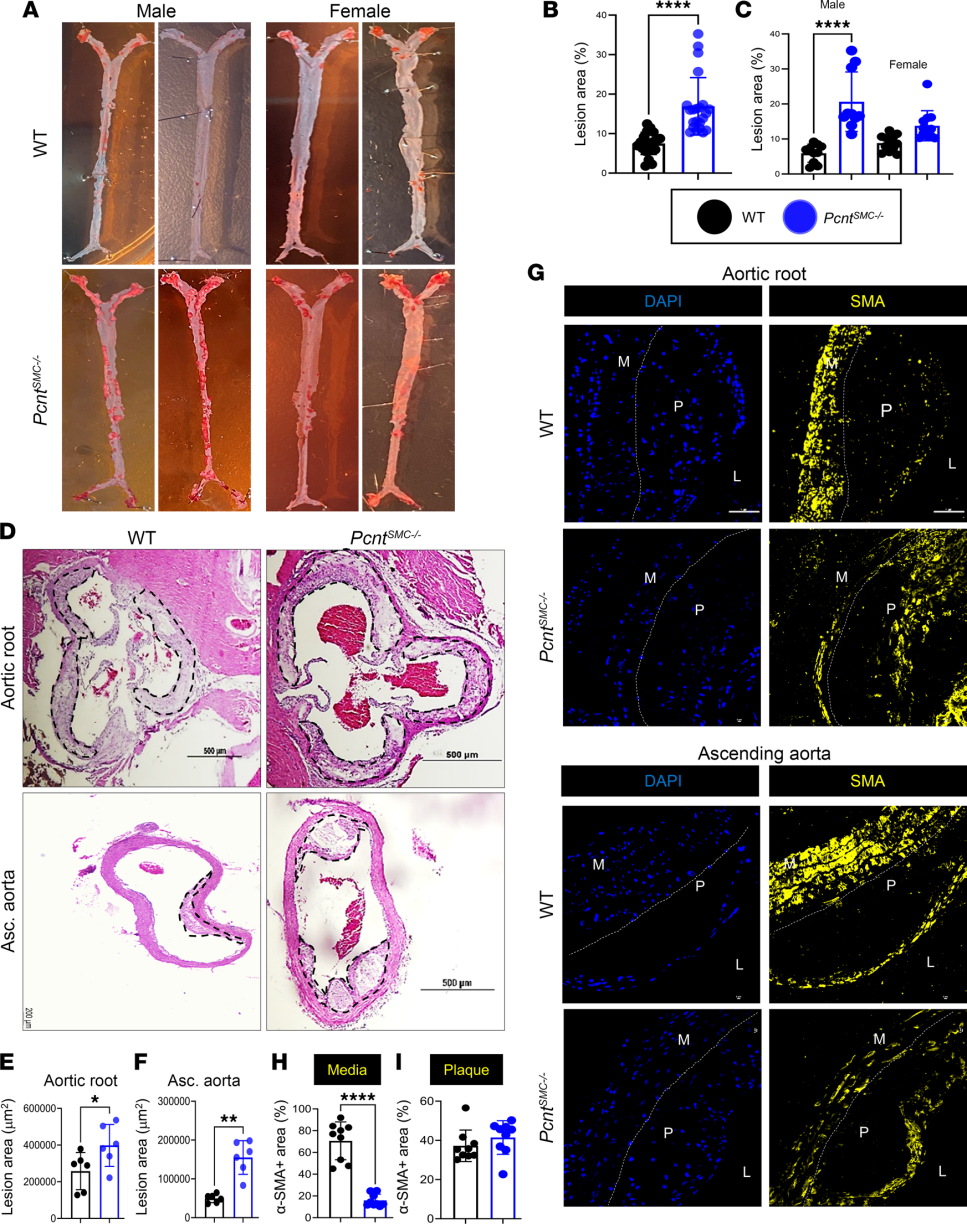
SMC-specific *Pcnt*-deficient mice have increased atherosclerotic plaque burden. (**A** and **B**) En face Oil Red O staining of aortas shows significantly increased plaque formation in hyperlipidemic *Pcnt^SMC–/–^* mice placed on 12 weeks of HFD, compared with similarly treated WT mice (*n* = 10–12 mice per genotype per sex, Mann-Whitney *U* test). (**C**) Only male *Pcnt^SMC–/–^* mice had significantly higher plaque burden compared with male WT mice, and female mice showed no statistically significant difference (*n* = 10 males and 12 females, 2-way ANOVA followed by Tukey’s multiple-comparison test). (**D**–**F**) H&E staining (**D**) demonstrates that male *Pcnt^SMC−/−^* mice had greater atherosclerotic lesion areas in both the aortic roots (**E**) and ascending aortas (**F**) (*n* = 6, by unpaired, 2-tailed Student’s *t* test with Welch’s correction). Scale bars: 200 μm (bottom left) and 500 μm (all others). (**G**–**I**) SMA staining of the aorta (**G**) reveals significantly fewer SMA^+^ cells in the medial layer (**H**) but not the plaque (**I**) of the aortic root (*n* = 9, Mann-Whitney *U* test). Error bars represent SD. *P < 0.05, **P < 0.01, ****P < 0.0001. L, lumen; M, medial layer; P, plaque. Scale bars: 5 μm.

Hyperlipidemia was induced in *Pcnt^SMC–/–^* and WT mice by injection of a single dose of adeno-associated virus expressing the pathological human D374Y gain-of-function mutant form of proprotein convertase subtilisin/kexin type 9 (AAV-*PCSK9^DY^*; 1.1 × 10^11^ viral particles) at 6 weeks of age, followed by a high-fat diet (HFD) initiated at 7 weeks of age (23, 24). After 12 weeks on the HFD, the weights of the male mice were significantly higher than female mice, but weight did not differ based on genotype (Supplemental Figure 1D). After 12 weeks on an HFD, plaque burden in the aortic root and ascending aorta of the *Pcnt^SMC–/–^* mice was significantly greater than WT mice based on Oil Red O staining (18.3% ± 7.4% versus 7.1% ± 2.8%; Figure 2, A and B). Male *Pcnt^SMC–/–^* mice had higher overall plaque burden compared with controls, but this difference did not reach statistical significance in female mice (*P* = 0.07) (Figure 2C). Hematoxylin and eosin (H&E) staining and quantification of plaques revealed significantly larger atherosclerotic lesions in both the aortic roots and ascending aortas of male *Pcnt^SMC–/–^* mice compared with WT mice (Figure 2, D–F). We did not perform histology for female mice since we did not see any significant difference in lesion area between genotypes, based on Oil Red O staining of whole aortas. Male WT and *Pcnt^SMC–/–^* mice had higher total cholesterol and lower triglycerides than their female counterparts, but there were no differences in lipid levels based on genotype (Supplemental Figure 1E).

**Figure 3:**
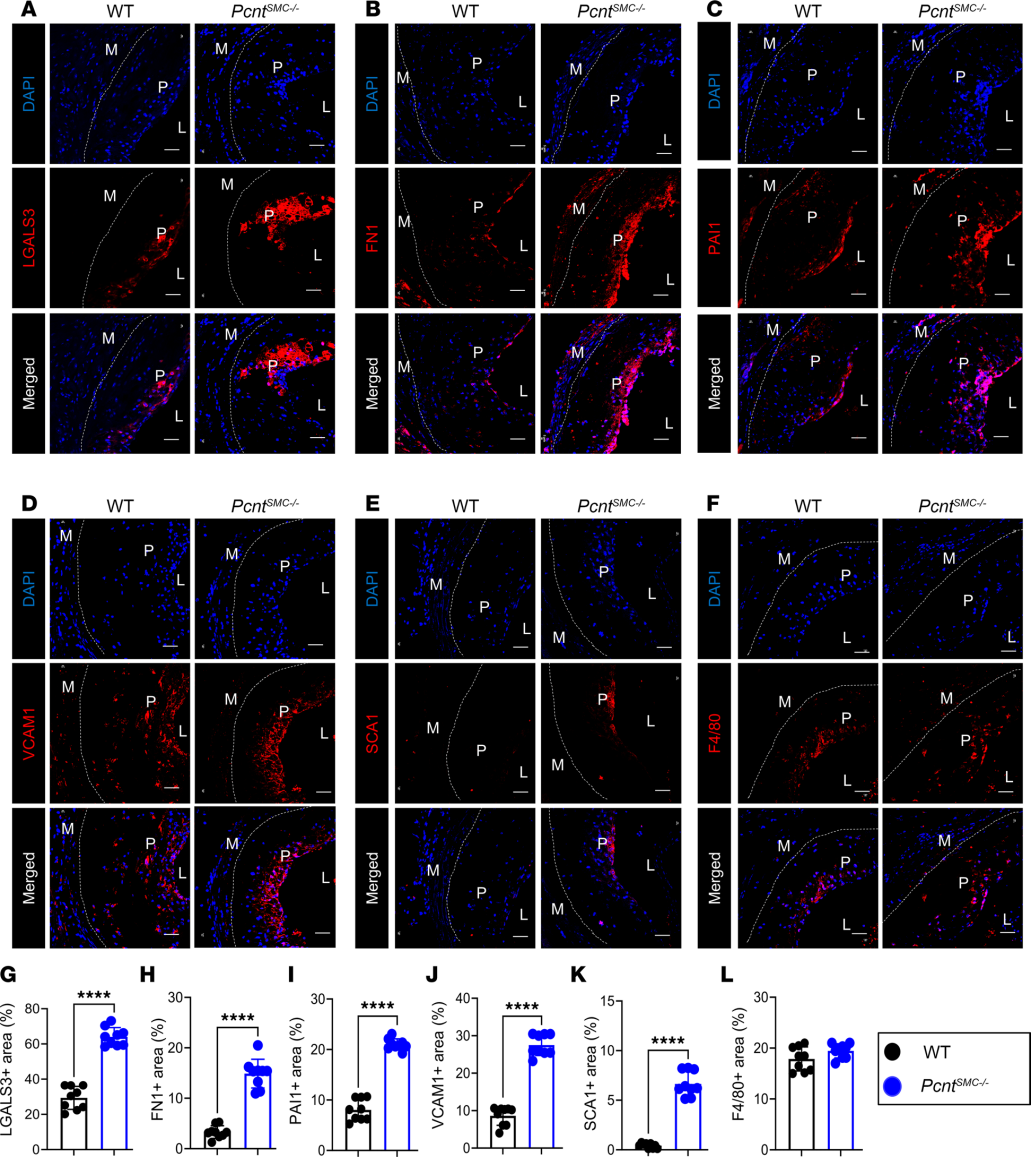
*Pcnt^SMC–/–^* mice show significantly increased expression of SMC modulation markers in aortic root lesions. (**A**–**E**) Immunohistochemical staining of aortic root sections for SMC modulation markers LGALS3, FN1, PAI1, VCAM1, and SCA1 and the differentiation marker SMA shows significantly higher staining for modulation markers and lower staining for SMA in *Pcnt^SMC–/–^* mice compared with WT mice. Nuclei were counterstained with DAPI (blue). (**F**) Immunohistochemical staining of aortic root sections for the macrophage marker F4/80 shows no change. (**G**–**K**) Quantification of the results in **A**–**E** (*n* = 9, quantification for LGALS3, FN1, PAI1, and SCA1 was analyzed by unpaired, 2-tailed Student’s *t* test followed by Welch’s correction and that for VCAM1 was analyzed by Mann-Whitney *U* test). (**L**) Quantification of the results in **F** (*n* = 9, unpaired, 2-tailed Student’s *t* test followed by Welch’s correction). Error bars represent SD. ****P < 0.0001. L, lumen; M, medial layer; P, plaque. Scale bar: 20 μm.

Immunofluorescent staining of aortic roots demonstrated significantly fewer SMA^+^ cells in the medial layer of *Pcnt^SMC–/–^* mice compared with WT mice, but similar levels in the atherosclerotic lesion (Figure 2, G–I). Cell density in the medial layer was significantly reduced in the aortic roots of *Pcnt^SMC–/–^* mice (Supplemental Figure 1F). Aortic plaques from *Pcnt^SMC–/–^* mice showed more staining for SMC modulation markers LGALS3, FN1, PAI1, VCAM1, and SCA1 compared with WT mice (Figure 3, A–E and G–K, and Supplemental Figure 2, A–E and G–K). SCA1^+^ cells are primarily in the fibrous caps of plaques in *Pcnt^SMC–/–^* aortic roots. However, no differences were observed in staining for a marker expressed only by macrophages (F4/80; Figure 3, F and L, and Supplemental Figure 2, F and L). Thus, the *Pcnt^SMC–/–^* mice have increased plaque burden that is associated with decreased SMCs in the medial layer and increased immunostaining in the plaque for markers of atherosclerosis-associated SMC modulation.

### Augmented phenotypic modulation of Pcnt^SMC–/–^ SMCs is due to increased HSF1 activation driving cholesterol biosynthesis and PERK signaling.

**Figure 4:**
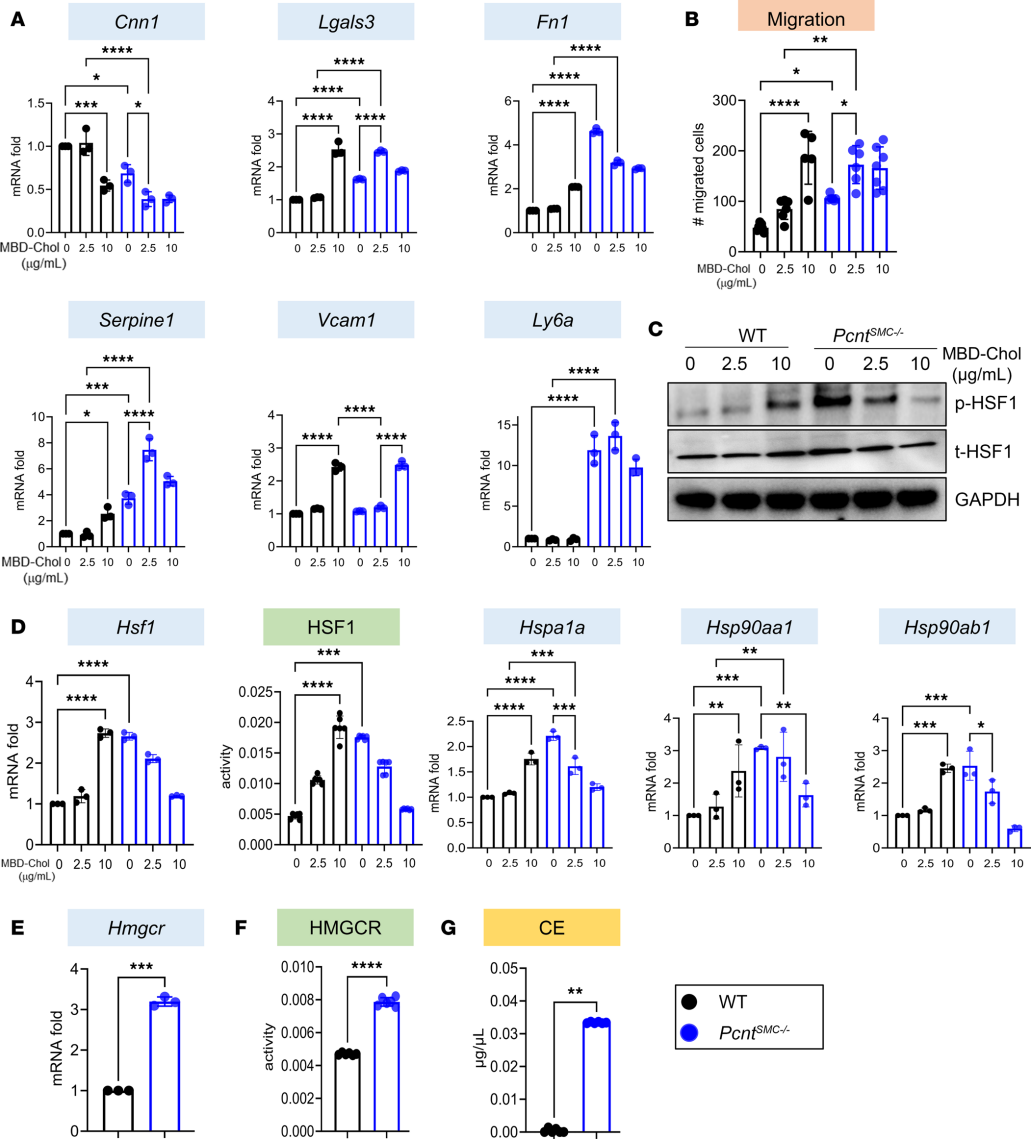
Augmented phenotypic modulation of *Pcnt^SMC–/–^* SMCs is due to increased HSF1 activation driving cholesterol biosynthesis. (**A**) Augmented SMC phenotypic modulation in *Pcnt^SMC–/–^* SMCs is evident from decreased mRNA expression of *Cnn1* and increased mRNA expression of modulation markers *Lgals3*, *Fn1*, *Serpine1*, and *Ly6a* either at baseline or with exposure to 2.5 μg/mL MBD-Chol in *Pcnt^SMC–/–^* SMCs, compared with 10 μg/mL MBD-Chol in WT SMCs, while *Vcam1* expression increases in both genotypes only with 10 μg/mL MBD-Chol. (**B**) *Pcnt^SMC–/–^* SMCs show increased migration by Transwell migration assay at baseline and with cholesterol exposure. (**C**) *Pcnt^SMC–/–^* SMCs exhibit increased levels of total and phosphorylated HSF1 (p-HSF1) at baseline. (**D**) HSF1 mRNA expression and luciferase activity and expression of HSF1 downstream targets *Hspa1a*, *Hsp90aa1*, and *Hsp90ab1* are also upregulated at baseline in *Pcnt^SMC–/–^* SMCs (all data in **A**–**D** passed normality and were analyzed by 2-way ANOVA followed by Tukey’s multiple-comparison test, except HSF1 activity, which was analyzed by Kruskal-Wallis test). (**E**–**G**) *Hmgcr* expression was significantly elevated in *Pcnt^SMC–/–^* SMCs (**E**), along with HMGCR enzymatic activity (**F**) and cholesteryl ester levels (**G**; data in **E**–**G** were analyzed by unpaired, 2-tailed Student’s *t* test followed by Welch’s correction). All gene expression data are representative of 3 independent experiments. Error bars represent SD. *P < 0.05; **P < 0.01; ***P < 0.001; ****P < 0.0001. CE, cholesteryl esters.

To determine whether pericentrin deficiency alters SMC phenotype, SMCs explanted from the ascending aortas of *Pcnt^SMC–/–^* and WT mice were assayed for expression of SMC modulation markers at baseline and after exposure to variable levels of exogenous free cholesterol complexed with methyl-β-cyclodextrin (MBD-Chol) (11). *Pcnt^SMC–/–^* SMCs were dedifferentiated based on the decreased expression of *Cnn1*, *Acta2*, and *Tagln* at baseline and with exposure to 2.5 μg/mL MBD-Chol, whereas the WT SMCs did not decrease expression of these genes until they were exposed to 10 μg/mL MBD-Chol (Figure 4A and Supplemental Figure 4A). The expression of atherosclerosis-associated modulation markers *Lgals3*, *Fn1*, and *Serpine1* (encoding PAI1) was increased at baseline and with 2.5 μg/mL MBD-Chol in the *Pcnt^SMC–/–^* SMCs, but these markers did not increase in the WT SMCs until 10 μg/mL MBD-Chol (Figure 4A). In contrast, *Ly6a* expression was greatly augmented in the mutant SMCs compared with WT SMCs and was not altered with cholesterol exposure, and *Vcam1* expression did not increase in either the *Pcnt^SMC–/–^* or WT SMCs until 10 μg/mL MBD-Chol (Figure 4A). *Pcnt^SMC–/–^* SMCs migrated faster than WT SMCs, both at baseline and with exposure to 2.5 μg/mL MBD-Chol, but did not demonstrate increased proliferation at baseline or with cholesterol exposure (Figure 4B and Supplemental Figure 4, B and C). There was no difference in apoptosis between WT and *Pcnt^SMC–/–^* SMCs, either at baseline or with cholesterol exposure (Supplemental Figure 4D).

Since loss of pericentrin in SMCs may activate cytosolic stress, total and phosphorylated HSF1 (Ser326), a marker for HSF1 activation, were assessed. Both were increased at baseline and with exposure to 2.5 μg/mL MBD-Chol in *Pcnt^SMC–/–^* SMCs, whereas the WT SMCs exhibited the same changes only at 10 μg/mL MBD-Chol (Figure 4C and Supplemental Figure 4E). Phosphorylated HSF1 translocates to the nucleus and binds to heat shock elements in the genome to activate transcription of heat shock proteins (HSPs) (25). *Pcnt^SMC–/–^* SMCs showed increased nuclear localization of HSF1, enhanced expression and transcriptional activity of HSF1, along with increased expression of its downstream targets *Hspa1a*, *Hsp90aa1*, and *Hsp90ab1* (Figure 4D and Supplemental Figure 4F). HSF1 activation increases cholesterol biosynthesis by increasing the expression of genes encoding cholesterol biosynthetic enzymes, including *Hmgcr* (13, 26). We found increased baseline *Hmgcr* expression and HMGCR activity, as well as increased expression of other cholesterol biosynthesis genes and elevated levels of cholesteryl esters in *Pcnt^SMC–/–^* SMCs compared with WT SMCs (Figure 4, E–G, and Supplemental Figure 4G). Taken together, these data show that *Pcnt*-deficient SMCs have increased HSF1 activation, cholesterol biosynthesis, and cholesterol ester levels, along with augmented expression of some markers of atherosclerosis-associated phenotypic modulation.

**Figure 5:**
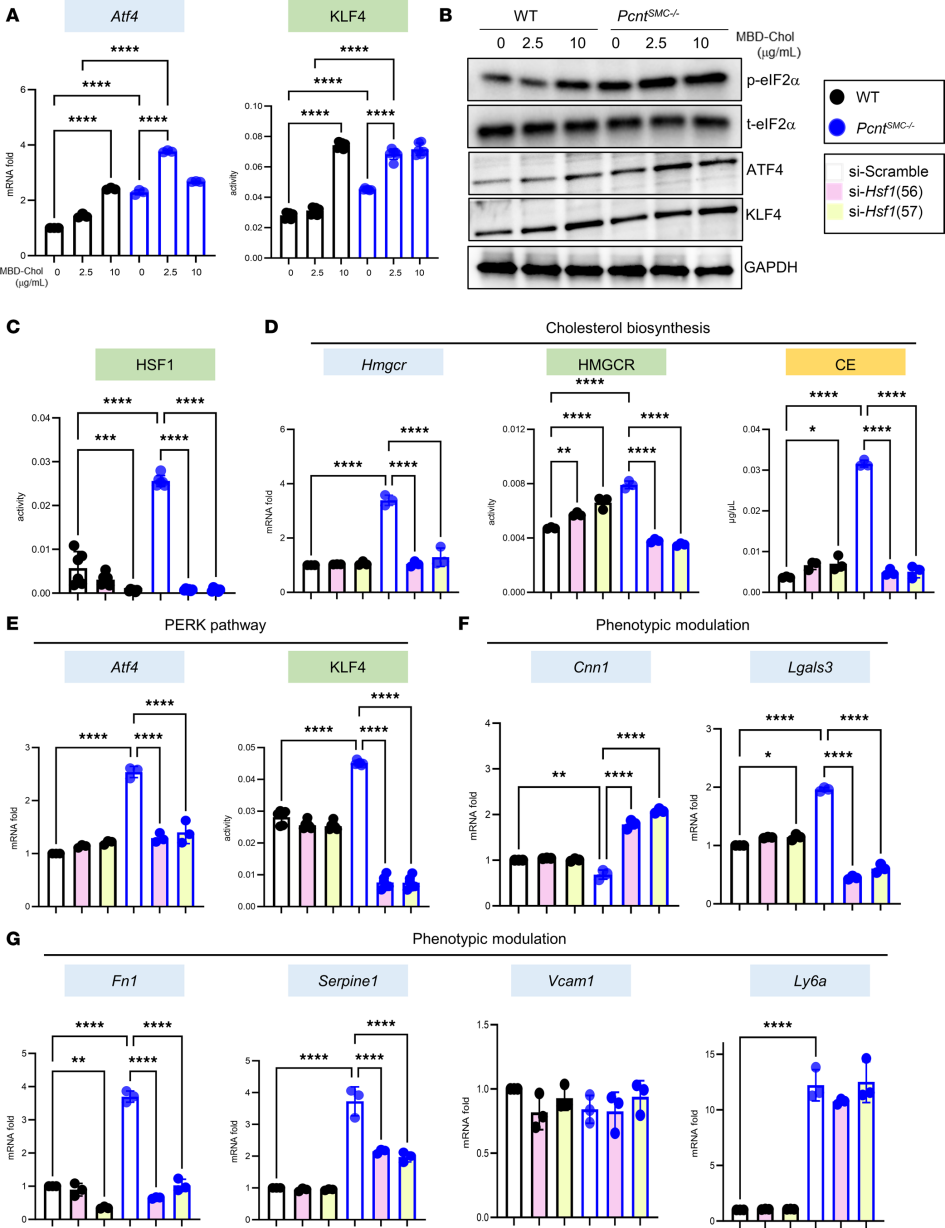
*Pcnt* deletion–induced augmented PERK signaling and SMC phenotypic modulation are HSF1 dependent. (**A** and **B**) *Pcnt^SMC–/–^* SMCs have increased *Atf4* expression and KLF4 luciferase activity (**A**), and increased levels of eIF2α phosphorylation, ATF4, and KLF4 (**B**), at baseline or with exposure to 2.5 μg/mL MBD-Chol in *Pcnt^SMC–/–^* SMCs, compared with 10 μg/mL MBD-Chol in WT SMCs. (**C**) HSF1 activity is significantly decreased in both WT and *Pcnt^SMC–/–^* SMCs following siRNA-mediated depletion of *Hsf1*. (**D**) *Hmgcr* expression, HMGCR enzymatic activity, and cholesteryl ester levels are significantly reduced in *Pcnt^SMC–/–^* SMCs following siRNA-mediated depletion of *Hsf1*. (**E**–**G**) siRNA-mediated depletion of *Hsf1* reduces activation of the PERK pathway (**E**), and phenotypic modulation (**F** and **G**) at baseline. All gene expression data are representative of 3 independent experiments. Multiple group comparisons were analyzed by 2-way ANOVA followed by Tukey’s multiple-comparison test. Error bars represent SD. *P < 0.05; **P < 0.01; ***P < 0.001; ****P < 0.0001. CE, cholesteryl esters.

We previously established that increased intracellular cholesterol activates PERK/ATF4/KLF4 signaling and is responsible for a component of atherosclerosis-associated phenotypic modulation of SMCs (5, 6, 11). *Pcnt^SMC–/–^* SMCs have activation of the 3 arms of the UPR, either at baseline or with exposure to 2.5 μg/mL MBD-Chol. PERK is activated based on increases in *Atf4* and *Klf4* expression and protein levels, KLF4 activity, and levels of phosphorylation of eIF2α in the *Pcnt^SMC–/–^* SMCs (Figure 5, A and B, and Supplemental Figure 5C). Evidence of IRE1α activation is based on augmented splicing of *Xbp1*, and ATF6 activation is based on increases of both total and cleaved ATF6 (Supplemental Figure 5, A and B). As we previously reported, WT SMCs activate these same UPR pathways only after exposure to 10 μg/mL MBD-Chol (11). A role of PERK signaling in SMC modulation was confirmed by exposing *Pcnt^SMC–/–^* SMCs to the PERK inhibitor, integrated stress response inhibitor (ISRIB), which blocks the expression of the phenotypic modulation markers, with the exception of *Vcam1* and *Ly6a* (Supplemental Figure 6). Two siRNAs verified to block *Hsf1* expression were used to confirm that HSF1 activation is upstream of both augmented cholesterol biosynthesis and PERK/ATF4/KLF4 activation in the *Pcnt^SMC–/–^* SMCs (Figure 5, C–E, and Supplemental Figure 5D). Knockdown of *Hsf1* also reversed baseline expression of markers of phenotypic modulation in *Pcnt^SMC–/–^* SMCs, with the exception of *Vcam1* and *Ly6a* (Figure 5, F and G).

### Inhibition of HMGCR with pravastatin prevents SMC phenotypic modulation and decreases plaque burden in Pcnt^SMC–/–^ mice.

**Figure 6:**
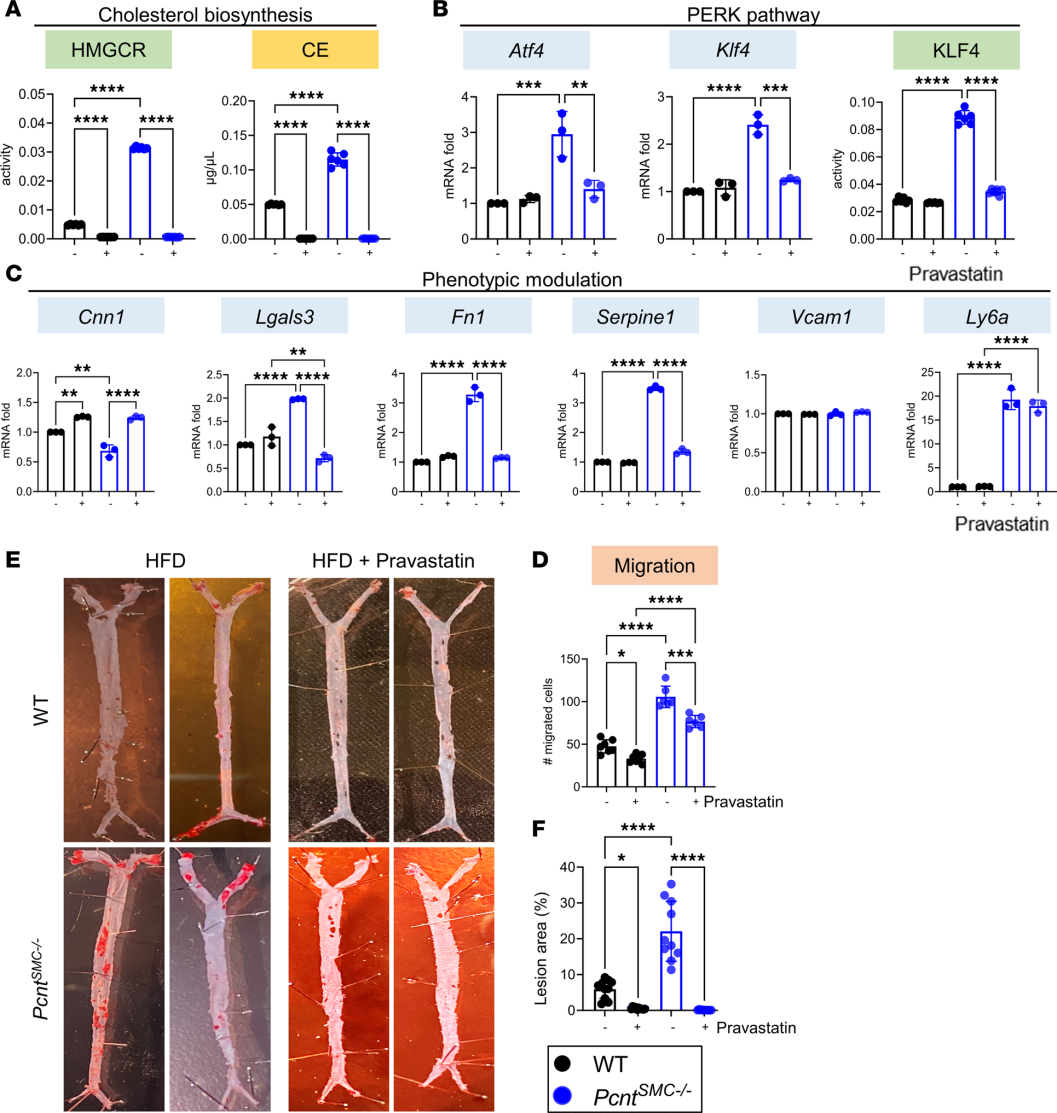
Treatment with the HMGCR inhibitor pravastatin reverses augmented SMC modulation and reduces the plaque burden in *Pcnt^SMC–/–^* mice. (**A**) Treatment with the HMGCR inhibitor pravastatin significantly reduces HMGCR activity and cholesteryl ester levels. (**B**–**D**) Pravastatin treatment suppresses baseline activation of the PERK pathway (**B**) and phenotypic modulation in *Pcnt^SMC–/–^* SMCs (**C**) and also decreases migration in both *Pcnt^SMC–/–^* and WT SMCs (**D**). (**E** and **F**) Treatment with pravastatin reduces plaque burden to similar levels in *Pcnt^SMC–/–^* and WT mice (*n* = 10 males). All gene expression data are representative of 3 independent experiments. Multiple group comparisons for both cellular and animal data were analyzed by 2-way ANOVA followed by Tukey’s multiple-comparison test. Error bars represent SD. *P < 0.05; **P < 0.01; ***P < 0.001; ****P < 0.0001. CE, cholesteryl esters.

After 72 hours of exposure to the HMGCR inhibitor pravastatin (250 nM), *Pcnt^SMC–/–^* and WT SMCs showed decreases in both HMGCR activity and levels of cholesteryl esters (Figure 6A). Pravastatin also inhibits PERK/ATF4/KLF4 signaling based on decreased levels of *Atf4* and *Klf4* expression, and decreased KLF4 activity (Figure 6B). Pravastatin also effectively prevented the increased migration (Figure 6D and Supplemental Figure 7A) and altered expression of modulation markers in *Pcnt^SMC–/–^* SMCs, with the exception of *Vcam1* and *Ly6a* (Figure 6C). Thus, HSF1-induced elevated HMGCR activation drives PERK/ATF4/KLF4 signaling and subsequent phenotypic modulation.

Finally, to determine whether the elevated baseline HMGCR activity in *Pcnt^SMC–/–^* SMCs was responsible for the increased atherosclerotic plaque burden observed in *Pcnt*-deficient mice, male WT and *Pcnt^SMC–/–^* mice were injected with AAV-*PCSK9^DY^* to induce hyperlipidemia at age 6 weeks, and then placed on an HFD and 50 mg/kg body weight/day pravastatin in the drinking water for 12 weeks, starting at age 7 weeks. As we found previously in the *Acta2^R149C/+^Apoe^–/–^* mice (11), pravastatin treatment led to death in all the female mice in both *Pcnt^SMC–/–^* and WT groups, 7 to 14 days after starting the treatment. En face Oil Red O staining of the aortas demonstrated that pravastatin effectively prevented plaque burden in both the *Pcnt^SMC–/–^* and WT mice, despite lowering serum lipid levels to similar extents in both genotypes (Figure 6, E and F, and Supplemental Figure 7B).

## Discussion

Biallelic loss-of-function variants in *PCNT*, encoding the large, conserved coiled-coil protein pericentrin, are the cause of MOPDII. Pericentrin is a key component of centrosomes, which have numerous and complex functions that involve cell cycle regulation and microtubule organization (18, 19, 27). The small stature and microcephaly in MODPII patients are hypothesized to result from disruption of cell division, leading to an overall reduction in the number of cells during growth (14). In contrast, decreased cell proliferation due to loss of pericentrin does not explain the premature vascular diseases in individuals with MOPDII, which include CAD and MMD. We previously determined that early-onset atherosclerosis associated with the *ACTA2* p.R149C variant results from activation of SMC cytosolic stress due to misfolding of the mutant SMA monomer (11). Therefore, we sought to determine whether loss of cytoplasmic pericentrin similarly activates SMC cytosolic stress to contribute to plaque burden (10, 11). We found that deletion of *Pcnt* from SMCs does indeed activate HSF1, which in turn increases the expression and activity of HMGCR, leading to increased endogenous cholesterol biosynthesis. The elevated intracellular cholesterol triggers ER stress and activates PERK/ATF4/KLF4 signaling, thereby augmenting SMC phenotypic modulation and increasing the atherosclerotic plaque burden. Thus, MOPDII is the second Mendelian disorder that predisposes to early onset atherosclerosis by augmenting SMC cytosolic stress, activating downstream HSF1/HMGCR/PERK signaling, and increasing SMC migration and atherosclerosis-associated phenotypic modulation. While the results reported here align closely with data from the *Acta2^R149C/+^* mouse model, *Acta2* is almost exclusively expressed in SMCs, and therefore, the pathogenic effect of *Acta2* variants can be assigned to altered SMC behavior (11, 28). In contrast, *Pcnt* is ubiquitously expressed and its loss in MOPDII potentially affects multiple cell types. The fact that SMC-specific deficiency of *Pcnt* augments the plaque burden reinforces the role of SMC cytosolic stress pathways in atherosclerosis, first demonstrated in our studies of the *Acta2^R149C/+^* mice. The embryonic lethality of global *Pcnt*-deficient mice limits its use to study atherosclerosis. Future studies using the *Pcnt*-floxed mice crossed with different tissue-specific *Cre* mice are needed to determine whether *Pcnt* deficiency in other cell types, including endothelial cells, macrophages, and other immune cells, also contributes to the atherosclerotic burden in individuals with MOPDII.

Based on en face aortic Oil Red O staining, male *Pcnt^SMC–/–^* mice showed significantly higher plaque burden compared with male WT counterparts, although this difference was not statistically significant for female mice (*P* = 0.07). While studies assessing the effect of sex on developing atherosclerosis in mouse models have reported contradictory results, multiple studies have demonstrated an atheroprotective role of estrogen in multiple mouse models (29–34). Additionally, previous studies have shown that AAV8-*PCSK9* injection results in lower *Pcsk9* mRNA expression in the liver and lower PCSK9 levels in the serum of female mice than male mice, and therefore male mice showed greater hyperlipidemia (35). Injecting WT C57BL/6 mice with a low dose of AAV8-*PCSK9* increased both serum PCSK9 and cholesterol levels only in male mice, whereas 3 times the original dose of AAV8-*PCSK9* was required to induce hyperlipidemia in the female mice (35). Based on these data, the sex-specific differences in *Pcsk9* expression in the liver and PCSK9 levels in the serum could contribute to the lower plaque burden in female mice.

The mixed genetic background of the *Pcnt^SMC–/–^* mice could potentially contribute to variability in plaque burden. To be certain that the *Pcnt^SMC–/–^* mice have increased plaque burden, we analyzed 10 male mice per genotype by Oil Red O staining, and 6 male mice per genotype by histopathology (higher overall number of mice than the AHA guidelines recommend). Both methods demonstrated that the *Pcnt^SMC–/–^* male mice had significantly higher plaque burden despite the mixed background.

MMD and intracranial aneurysms are identified in two-thirds of individuals with MOPDII, with one-third of the patients having both diseases (36). These cerebrovascular diseases are partially responsible for the 50% mortality of MOPDII patients by the age of 25 years. One-fifth of MOPDII cases have CAD, with a slightly older age of onset than the cerebrovascular diseases, and other occlusive lesions identified in the renal, femoral, and external carotid arteries may also be due to atherosclerosis (15, 16, 36). Here, we found that CAD in patients with MOPDII has some typical atherosclerotic plaque pathology, but also has marked depletion of SMCs from the wall of the arteries and increased elastin in the adventitial layer. Clonal expansion of SMCs in the intima has been shown to contribute to atherosclerotic plaques (37). However, based on the increased migration in the absence of augmented proliferation of *Pcnt^SMC–/–^* SMCs, SMC migration from the media to the intima may have a more significant effect on the plaque burden in this model system. This is underscored by the reduced medial cell density observed in the coronary arteries from patients with MOPDII and the hyperlipidemic *Pcnt^SMC–/–^* aortas. Additionally, the increased elastosis in the adventitial layer associated with plaque formation leads us to speculate that migration of SMCs may also occur into the adventitial layer.

Both MMD and CAD lesions involve migration of SMCs from the media into the intima, but atherosclerotic lesions uniquely contain cholesterol crystals and cholesterol-laden macrophages, whereas moyamoya lesions show lumens filled with neointimal cells that stain positive for SMC markers (8, 9, 38, 39). Thus, MMD is most likely not associated with SMC atherosclerosis-associated phenotypic changes except for increased migration. HSF1 is an established tumor-promoting signaling factor that is involved in cancer cell migration, and therefore HSF1 driving SMC migration into the lumen may also contribute to MMD lesions (40). The accumulating risk factors for CAD in patients with MOPDII, such as hypercholesterolemia, hypertension, and chronic kidney disease, most likely contribute to the slightly later-onset atherosclerosis-associated SMC phenotypic modulation and CAD in these patients. In fact, we found that migration and the expression of some modulation markers of *Pcnt^SMC–/–^* SMCs increased even further with exposure to low levels of exogenous cholesterol. However, the role of HSF1-driven migration in MMD pathogenesis in individuals with MOPDII needs to be further studied. We speculate that if there is a similar loss of SMCs in the medial layer of the cerebrovascular arteries due to augmented migration into the neointima, it may contribute to intracranial aneurysm formation.

Similar to our observation in *Acta2^R149C/+^* SMCs, *Pcnt*-deficient SMCs have significantly higher HSF1 activation at baseline than WT SMCs, and this activation progressively decreases with cholesterol exposure in the mutant SMCs but increases in the WT SMCs (11). In *Acta2^R149C/+^* SMCs, the baseline activation of HSF1 results from misfolding of mutant SMA, and the progressive decrease in HSF1 activation with cholesterol treatment is attributed to decreasing SMA levels as the cells dedifferentiate. In contrast, *Pcnt* expression should not change with cholesterol exposure, and HSF1 activation should remain high, especially given that the mutant SMCs remain viable when exposed to 10 μg/mL MBD-Chol. *Pcnt* loss leads to cell cycle arrest and ataxia-telangiectasia-mutated-and-Rad3-related kinase/checkpoint kinase 1 (ATR/CHK1) pathway dysfunction, and HSF1 is known to form a complex with DNA, ATR, and CHK1 to control cell cycle progression (41, 42). Studies have demonstrated that cell cycle progression is dependent on active cholesterol biosynthesis (43). Therefore, it is possible that cell cycle–arrested *Pcnt^SMC–/–^* SMCs upregulate HSF1 at baseline to enhance HMGCR expression and activity to increase intracellular cholesterol biosynthesis, which in turn accelerates cell cycle progression. Exposure to exogenous cholesterol relieves this cell cycle arrest in *Pcnt^SMC–/–^* SMCs, and thereby reduces cytosolic stress and HSF1 activation.

The SMC phenotypic modulation markers used in this study are regulated by the HSF1/HMGCR/PERK signaling pathway, with the exception of *Vcam1* and *Ly6a* (encodes stem cell antigen 1, SCA1). Both WT and *Pcnt^SMC–/–^* SMCs increase *Vcam1* expression when exposed to 10 μg/mL MBD-Chol and we previously confirmed that *Vcam1* is an SMC modulation marker that is induced by exposure to high level of cholesterol and is not dependent on PERK signaling, but rather an unidentified signaling pathway (6). In contrast, *Ly6a* expression is increased at baseline in the *Pcnt^SMC–/–^* SMCs but not WT, and its expression is not altered with cholesterol exposure. Neither *Hsf1* depletion nor PERK inhibition (which in turn inhibits KLF4 activation) decreases *Ly6a* expression, indicating that *Ly6a* expression is regulated by pathways other than those investigated in this study (44, 45). SCA1 has been previously shown to be involved in cellular migration, and the significantly higher baseline expression of *Ly6a* could contribute to the increased migration of *Pcnt^SMC–/–^* SMCs (11, 46). Pravastatin treatment reduces the augmented migration in *Pcnt^SMC–/–^* cells, but does not reduce it to the level of WT SMCs, which could be attributed to the continued high *Ly6a* expression in these cells.

An unbiased method to identify whether genes known to promote atherosclerosis are upregulated in *Pcnt^SMC–/–^* SMCs would be to perform bulk RNA-seq or scRNA-seq, and reconcile these data with loci identified in genome-wide association studies (GWAS) for CAD. There are approximately 300 GWAS loci reported to be associated with CAD in humans (47). Among the genes identified in this study and predicted to be involved at a GWAS locus, we only identified KLF4 as a positive hit; *Klf4* expression, protein level, and transcriptional activation are significantly increased in *Pcnt*-deficient SMCs compared with WT SMCs and KLF4 is associated with a GWAS locus (11). Future studies will focus on assessing the SMC transcriptomics profiles of *Pcnt^SMC–/–^* mice in a pure C57BL/6 background with an SMC lineage tracer to determine whether genes with altered expression are associated with CAD GWAS loci.

For *ACTA2* and *PCNT* pathogenic variants, SMC cytosolic stress that activates HSF1/HMGCR/PERK signaling contributes to early-onset atherosclerosis, raising the possibility that SMC cytosolic stress underlies premature atherosclerosis associated with other genetic diseases. These conditions include the following: Williams-Beuren syndrome, a disorder caused by a hemizygous deletion in a region of chromosome 7q11.23; hyperhomocysteinemia, due to loss-of-function variants in cystathionine-β-synthase (*CBS*) or methylenetetrahydrofolate reductase (*MTHFR*) genes; pseudoxanthoma elasticum (caused by defects in *ABCC6*); Hutchinson-Gilford progeria syndrome (HGPS) due to overexpression of progerin, a truncated form of lamin A; and Werner syndrome due to pathogenic variants in *RECQL2* (9, 48–51). Limited data from a HGPS mouse model provide evidence that HSF1/HMGCR/PERK signaling may contribute to early atherosclerosis in patients with HGPS. HGPS patients have premature physiological aging and childhood-onset atherosclerosis, including myocardial infarction and stroke, in the absence of hypercholesterolemia (51–53). SMC-specific overexpression of progerin in mice exacerbates atherosclerosis and is associated with increased ER stress and PERK signaling in SMCs. Furthermore, atherosclerotic burden can be reduced by treating these mice with an inhibitor of ER stress, tauroursodeoxycholic acid (54, 55). Since activation of PERK may be downstream of progerin-induced cellular stress, SMC activation of HSF1/HMGCR/PERK may contribute to the increased risk for atherosclerosis in individuals with HGPS.

Given that the mechanistic pathway identified here to drive augmented atherosclerosis with pericentrin loss is dependent on SMC cholesterol biosynthesis, statins provide a targeted therapy to block the pathway specifically in patients with MOPDII. Statins are only administered to hypercholesterolemic patients with MOPDII, and our data indicate that statins should be considered for all MOPDII patients, whether they have elevated plasma lipids or not (17). The MOPDII cases reported here had statins started in their early 20s since they had hyperlipidemia, and both died of CAD a few years later, which raises the question as to whether they had high burden of CAD when the statins were started. Given that recent studies indicate that coronary artery atheromas develop in people in their 40s, the coronary artery changes in MOPDII most likely occur very early, but loss of pericentrin in other cell types may also contribute to atherosclerotic burden (56). How early to start statins in children with MOPDII is not known. HSF1-driven SMC modulation includes phenotypic changes that mimic osteogenic cells, which are responsible for the calcium deposits in plaques (4, 11). Therefore, cardiac calcium imaging in patients with MOPDII may be an early diagnostic marker of CAD development and thus inform when to start statin tratment. Importantly, statin therapy should be more effective in treating the premature CAD associated with MOPDII than *PCSK9* inhibitors since statins directly target a critical enzyme involved in the augmented SMC modulation, specifically HMGCR and cholesterol biosynthesis in SMCs. In contrast, *PCSK9* inhibitors enhance recycling of LDLR by inhibiting targeting of LDLR to the lysosome for degradation (57). Finally, we found that statins also effectively decrease *Pcnt^SMC–/–^* SMC migration, and if SMC migration contributes to moyamoya lesions in individuals with MOPDII, statins may help to prevent or attenuate MMD in these patients.

Statins are not a component of the current treatment recommendations for preventing vascular diseases in patients with MOPDII unless those patients have hyperlipidemia, and the data presented here indicate that statins should be considered for all patients with MOPDII to prevent atherosclerosis. We hypothesize that statin treatment started at an early age, and even in the absence of hyperlipidemia, would be helpful for patients with MOPDII to prevent early-onset CAD and may even decrease the risk for MMD (17). The molecular mechanism uncovered by this study reinforces the critical contribution of SMCs in the pathogenesis of atherosclerosis and further emphasizes SMC cytosolic stress and HSF1 activation as a pathway driving atherosclerotic plaque formation independently of plasma cholesterol levels.

## Methods

### Analyses of patient tissue.

Coronary artery tissue was obtained at autopsy and stained with H&E and Movat pentachrome and immunostained with an anti-SMA antibody (MilliporeSigma, A5228).

### Generation of SMC-specific Pcnt-knockout mice.

Embryonic stem cell clones in a C57BL/6 background with the targeted *Pcnt* allele (*Pcnt^tm1a(EUCOMM)Wtsi^*; clones EPD0724_5_F03, B04, and A03) were purchased from the European Conditional Mouse Mutagenesis program (https://www.mousephenotype.org/about-impc/about-ikmc/eucomm/) (58). The targeting vector was designed to contain a 5391-bp 5′-targeting arm and *LacZ* and neo expression cassettes, which were flanked by 2 *Frt* sites. In addition, it contained a 1499-bp mouse *Pcnt* fragment (spanning exons 17 and 18) that was flanked by 2 *LoxP* sites and a 4438-bp 3′-targeting arm allowing for Cre-mediated conditional deletion of the *Pcnt* gene between exons 17 and 18, leading to an out-of-coding frame shift generating a premature stop codon and a loss-of-function allele. The embryonic stem cell clones were microinjected into C57BL/6 blastocysts. Male chimeric founder mice were bred with albino C57BL/6 females. Male chimeric mice with proven germ line transmission were then bred into 129S4/SvJaeSor-*Gt*(*ROSA*)*26Sor^tm1(FLP1)Dym^*/J mice (stock 003946, The Jackson Laboratory) to remove the *Frt*-flanked *LacZ* and neo cassettes. To study the development of atherosclerotic disease in vivo, we utilized a constitutive SM22α-Cre in conjunction with our *Pcnt^fl/fl^* mice to enable knockout of *Pcnt* early in development. *Pcnt^fl/fl^* and *SM22*α*Cre^+/–^* mice [B6.Cg-Tg(*Tagln*-Cre)1Her/J, strain 017491) were obtained from The Jackson Laboratory. The *SM22*α*Cre^+/–^* *Pcnt^fl/+^* mice obtained from crossing the above 2 strains were backcrossed into *Pcnt^fl/fl^* mice to obtain *SM22*α*Cre^+/–^* *Pcnt^fl/fl^* mice (designated as *Pcnt^SMC–/–^*). *SM22*α*Cre^–/–^* *Pcnt^fl/fl^* littermates were used as controls and are referred to as WT.

### AAV-PCSK9^DY^ injection, HFD, and pravastatin treatment.

At 6 weeks of age, both male and female *Pcnt^SMC–/–^* and WT mice were injected with a single dose of AAV-*PCSK9^DY^* containing 1.1 × 10^11^ viral particles (packaged at the University of North Carolina Vector Core, Chapel Hill, North Carolina, USA) into a retro-orbital vein. At 7 weeks of age, the mice were placed on HFD (TD.88137, Envigo), with or without 50 mg/kg pravastatin via drinking water (males only) and maintained for 12 weeks (23, 24). For pravastatin treatment, only male mice (*n* = 10 per treatment per group) were used to complete the trial since all the female mice died within 1–2 weeks of starting the treatment. Similar to our previous observation, all the female mice died within 1-2 weeks of initiating the trial (11). Necropsy of the female mice revealed enlarged and darkened livers along with dried blood around their noses. The mice also appeared to have suffered from weight loss, but since rigor mortis had set in by the time the mouse carcasses were discovered, our ability to accurately determine their weight was limited.

### Lipid profile analysis.

Total cholesterol and triglycerides were analyzed in the serum of *Pcnt^SMC–/–^* and WT mice 12 weeks after they were placed on the HFD using fast performance liquid chromatography at the Baylor College of Medicine Mouse Metabolism and Phenotypic Core, as described previously (*n* = 10–12 mice per sex per genotype per treatment) (59).

### En face Oil red O staining of aortas.

Aortas were opened longitudinally to expose the lumen, washed with 60% isopropanol for 30 seconds, and stained with Oil Red O solution (0.3% in 60% isopropanol) using standard protocols. Ten to 12 aortas were analyzed per genotype per sex (10 males and 12 females per genotype), according to the guidelines recommended by the American Heart Association (24). Plaques were quantified using ImageJ software (NIH) and are expressed as percentage area covered in plaque relative to the area of the entire aorta. Analysis was performed by 2 blinded individuals.

### Histopathology.

Paraffin cross sections (5 μm) from fixed tissues (root and ascending aorta) were stained with H&E (*n* = 6) or used for immunohistochemistry according to established protocols (*n* = 9). H&E-stained sections were imaged using a Zeiss LSM 800 microscope. Five to 10 randomly chosen fields were imaged per sample for each stain. Lesion areas were measured by 2 blinded individuals using ImageJ. For calculation of cellularity in the tunica media, cells were counted manually by 2 blinded individuals and ImageJ software was used to determine the areas of the field of view, and cellularity was expressed as number of cells/mm^2^. For immunostaining, formalin-fixed paraffin-embedded (FFPE) aortic sections from experimental and control mice were deparaffinized, rehydrated, and then subjected to antigen retrieval using sodium citrate buffer (pH 6.0) at 98°C for 20 minutes. The tissues were then permeabilized with Tris-buffered saline (TBS) containing 0.025% Triton X-100 and blocked for 1.5 hours with 5% BSA in TBS. The sections were then incubated with anti-SMA antibody in combination with any one of the following antibodies: anti-F4/80, anti-LGALS3, anti-FN1, anti-PAI1, anti-VCAM1, and anti-SCA1. Antibody information is provided in Table 1. Following overnight incubation at 4°C, the slides were washed with TBS containing 0.01% Tween 20 (TBST) and incubated with anti-goat Alexa Fluor 647 with anti-rabbit or anti-rat Alexa Fluor 594 for 1 hour at room temperature. The tissues were then washed again with TBST and mounted with ProLong Diamond Antifade Mountant (Invitrogen), following which the slides were allowed to dry for 24 hours in the dark. Imaging was performed using a Nikon A1 Confocal Laser Microscope at the UTHealth Center for Advanced Microscopy. Negative control staining was performed using normal goat, rat, or rabbit IgG (Supplemental Figure 3).

### SMC explants and cholesterol treatment.

Aortic SMCs were explanted from the ascending aortas of *Pcnt^SMC–/–^* and WT mice as described previously (60). SMCs were treated with indicated amounts of free cholesterol complexed with methyl-β-cyclodextrin (MBD-Chol, MilliporeSigma) with and without ISRIB (Tocris Bioscience) in Dulbecco’s modified Eagle medium (DMEM) containing high glucose (Cellgro), 10% FBS (Gibco), 1% antibiotic/anti-mycotic (MilliporeSigma), and 0.2% BSA (Thermo Fisher Scientific) with and without drugs as indicated for 72 hours at 37°C and 5% CO_2_.

### RNA extraction and qRT-PCR.

Total RNA was isolated from cultured SMCs using a PureLink RNA Mini kit (Thermo Fisher Scientific), followed by quantification using a Nanodrop (Thermo Fisher Scientific). cDNA was synthesized using QScript reagent (Quantabio). qRT-PCR was performed using TaqMan chemistry for contractile genes (Applied Biosciences) and SYBR Green (MilliporeSigma) for all other genes using master mixes obtained from Quantabio. *Gapdh* and *18S* rRNAs were used as endogenous controls for TaqMan and SYBR reactions, respectively. Please refer to Supplemental Table 2 for details of qPCR primers used.

### Protein extraction, subcellular fractionation, and Western blotting.

Cold RIPA buffer was prepared with protease inhibitor cocktail (MilliporeSigma) and phosphatase inhibitor cocktails (MilliporeSigma). Following indicated treatments, cells were collected via scraping and incubated in lysis buffer for 15 minutes followed by 15-second sonication. Crude lysates were then cleared via centrifugation at top speed for 10 minutes at 4°C. For subcellular fractionation, cells were harvested by trypsinization and then fractionated into nuclear and cytosolic fractions using a CelLytic NuCLEAR Extraction kit (MilliporeSigma, NXTRACT-1KT). Nuclear lysates were sonicated using a Branson sonicator at 60% power for 20 seconds at 4°C before estimating the protein concentration. Bradford assay was performed per protocol (Bio-Rad Laboratories) and protein concentrations quantified. For pericentrin, 60–80 μg of protein was resolved in a 4% gel (ProtoGel National Diagnostics). For all other samples 10–40 μg of protein was resolved in 4%–0% TGX gels (Bio-Rad Laboratories). Protein was then transferred to PVDF membranes (MilliporeSigma), blocked with 5% dry milk (or 5% BSA for detecting phosphorylated proteins, ATF4, and pericentrin) in TBST, and the membranes probed with different antibodies. Bands were visualized by chemiluminescent substrate (GE Healthcare) on a Bio-Rad imager. Band intensities on the immunoblots were quantified using ImageJ software. Detailed information about antibodies is provided in Table 1.

### Cell proliferation assay.

Cellular proliferation was measured using a Click-iT Plus EdU Alexa Fluor 647 Flow Cytometry Assay Kit (Thermo Fisher Scientific), according to manufacturer’s instructions. Briefly, 5 hours prior to harvesting, 5-ethynyl-2′-deoxyuridine (EdU) was added to the cells at a final concentration of 10 μM. The cells were harvested by trypsinization, followed by fixation, permeabilization, incubation with EdU detection cocktail, and analyzed by flow cytometry using a BD LSR Fortessa instrument at the UTHealth Flow Cytometry Service Center.

### Transwell migration assay.

*Pcnt^SMC–/–^* and WT cells were plated on the upper layer of a Transwell cell culture insert containing a permeable membrane (Corning Inc.), allowed to attach overnight, and then treated with MBD-Chol for 72 hours. The cells were then washed with PBS, methanol, and distilled water, followed by staining with NucBlue (Thermo Fisher Scientific). The permeable membranes containing the migrated cells were excised, mounted on glass slides using Permount (Thermo Fisher Scientific), covered with coverslips, and sealed with clear nail polish. The cells were then imaged using filters for DAPI on a Zoe Fluorescent Cell Imager (Bio-Rad Laboratories). The experiment was performed in triplicate using independent samples and 4 randomly chosen fields were imaged per sample each time. The migrated cells were counted using ImageJ software.

### Luciferase assays for HSF1 and KLF4 activation.

Luciferase assays for transcriptional activation of HSF1 and KLF4 were performed by transfecting 25,000 SMCs (in triplicate) with 250 ng heat shock element (HSE) or KLF4 Cignal Reporter plasmid (Qiagen) using Lipofectamine reagent (Life Technologies), followed by treating the cells with cholesterol and finally measuring the firefly and Renilla luciferase luminescence using a Dual Luciferase Assay Kit (Promega), as per the manufacturer’s instructions. Luciferase activity is expressed as the ratio between the firefly and the Renilla luciferase luminescence.

### HMGCR.

HMGCR activity in explanted WT and *Pcnt^–/–^* SMCs was determined using an HMGCR activity assay kit (Abcam, ab204701) according to the manufacturer’s instructions. Briefly, cell lysates were cleared by centrifugation and protein concentration in the lysates was estimated using Bradford assay. Immediately after adding the reaction cocktail (reaction buffer, NADPH, and HMG-CoA), absorbance was measured in kinetic mode at 340 nm and the reaction was followed for 20 minutes, collecting readings every 2 minutes. Absorbance values at 2 time points in the linear range were used to calculate the enzymatic activity per mg protein.

### Cholesteryl ester formation assay.

A Cholesterol/Cholesteryl Ester Quantitation Assay kit (Colorimetric/Fluorometric, Abcam, ab65359) was used to quantify cholesteryl ester formation in explanted SMCs. SMCs (100,000 per genotype) were plated in complete growth media in each well of a 6-well plate overnight. The media were replaced with high-glucose DMEM with 10% FBS, 1% antibiotics, and 0.2% BSA, and the cells were incubated for 72 hours. SMCs were harvested by trypsinization and collected by brief centrifugation. Lipids were extracted from the cell pellets by vortexing them in a mixture of chloroform, isopropanol, and NP-40. Following centrifugation, the organic phase was air dried at 50°C to remove the chloroform. The solid residue was used to perform a colorimetric assay to determine cholesteryl ester content according to the manufacturer’s instructions. Total and free cholesterol concentrations were calculated using a standard curve and the difference between the 2 values was the cholesteryl ester concentration.

### Statistics.

All data shown are expressed as mean ± SD. Data were tested for normality using GraphPad Prism software version 9.2.0. The following data passed normality and were analyzed using unpaired, 2-tailed *t* test with Welch’s correction: atherosclerotic lesion size using Oil Red O (genotypes were not separated by sex), HDL, LDL, VLDL, total cholesterol, and quantification of aortic root lesions by histology. Atherosclerotic lesion size using Oil Red O when genotypes were separated by sex, along with qPCR, luciferase assays, and migration data using explanted cells treated with MBD-Chol also passed normality and were analyzed by 2-way ANOVA, followed by Tukey’s multiple-comparison test. The following data did not pass normality and were analyzed using Mann-Whitney *U* test: triglyceride levels, lesion quantification in the ascending aorta, quantification of foam cells, and quantification of α-SMA staining. A *P* value of less than 0.05 was considered statistically significant.

### Study approval.

The study protocol was approved by the Institutional Review Board of the University of Texas Health Science Center at Houston and the patient or family members provided written informed consent to be included in these studies. All animal studies were performed according to protocols approved by the Institutional Animal Care and Use Committee at the University of Texas Health Science Center at Houston (AWC-21-0126) and in accordance with the NIH *Guide for the Care and Use of Laboratory Animals* (National Academies Press, 2011).

### Data availability.

The numerical data used to generate the graphs in this study are provided in the Supporting Data Values file. Non-numeric data presented in the article are available from the corresponding author upon request.

## Author contributions

DMM, SM, AC, CSK, and PG conceptualized the study. JMW and SM generated the experimental animals. SM performed the animal experiments and SM, AC, and CSK performed cellular experiments. SM and AC performed the data analysis. LMB, JMW, and DMM analyzed the patients’ pathology slides. SM, AC, PG, CSK, LMB, and DMM drafted and edited the manuscript. DMM acquired funding for the study. The order of equal first authorship was determined based on the fact that SM performed all the animal experiments and some cellular experiments, while AC performed the majority of the cellular experiments.

## Supplementary Material

Supplemental data

Supporting data values

## Figures and Tables

**Table 1:**
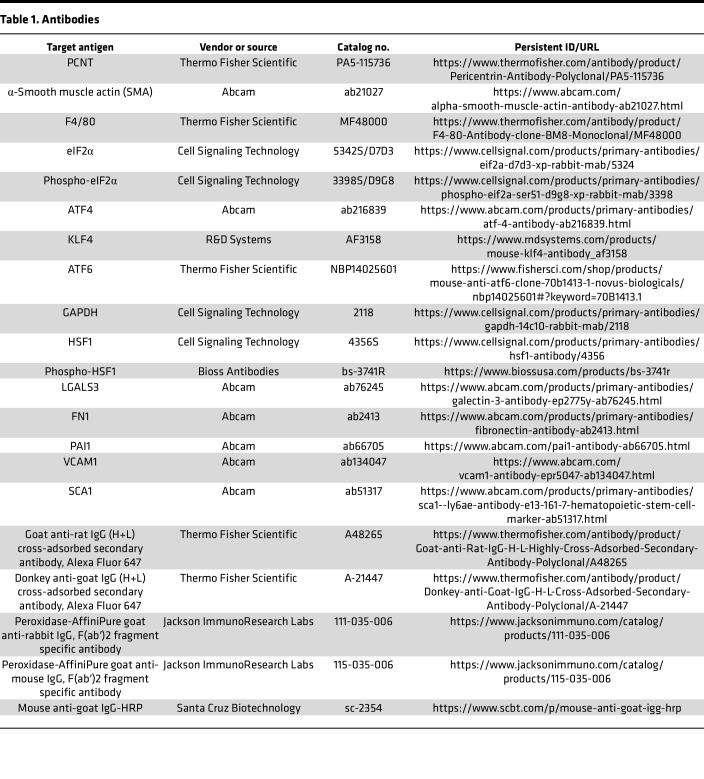
Antibodies

## Version 1 11/08/2023

Electronic publication
