# Supplementary material for: Pericentrin deficiency in smooth muscle cells augments atherosclerosis through HSF1-driven cholesterol biosynthesis and PERK activation
Source: JCI Insight. 2023 Nov 8;8(21):e173247. doi: 10.1172/jci.insight.173247 (PMC10721278; doi:10.1172/jci.insight.173247)
Supplement: Supplemental data [file jciinsight-8-173247-s067.pdf]

Supplemental Material for:

Pericentrin deficiency in smooth muscle cells augments atherosclerosis through HSF1-driven cholesterol biosynthesis

Suravi Majumder, Abhijnan Chattopadhyay, Jamie M. Wright, Pujun Guan, L. Maximillian Buja, Callie S. Kwartler, Dianna M. Milewicz.

This file contains:

Supplemental tables 1-2

Supplemental figures 1-7

Supplemental Table 1

Animals

**Species**	**Vendor or Source**	**Background Strain**	**Sex**	**Persistent ID / URL**
Mus musculus Pcnt$^{floxed/floxed}$	This paper	C57BL/6J and 129S4/SvJaeSor	M/F	N/A
Mus musculus STOCK B6.Cg-Tg(Tagln-cre)1Her/J	The Jackson Laboratory	C57BL/6J	M/F	https://www.jax.org/strain/017491

Supplemental Table 2

SYBR Green primers for qPCR

Gene	Forward primer (5’ -> 3’)	Reverse primer (5’ -> 3’)
18s rRNA	GTAACCCGTTGAACCCCATT	CCATCCAATCGGTAGTAGCG
Atf4	GGGTTCTGTCTTCCACTCCA	AAGCAGCAGAGTCAGGCTTTC
Fdps	GGAGGTCCTAGAGTACAATGCC	AAGCCTGGAGCAGTTCTACAC
Fn1	CGAGGTGACAGAGACCACAA	CTGGAGTCAAGCCAGACACA
Hmgcr	TGTCCCCACTATGACTTCCC	TCGGTGGCCTCTAGTGAGAT
Hmgcs1	TGGCAGGGAGTCTTGGTACT	TCCCACTCCAAATGATGACA
Hsp90aa1	AATGCTTAGAACTATTTACTGAACTAGCAGAA	GTCCTCGTGAATTCCAAGCTTT
Hsp90ab1	GCGCACGCTGACTTTGGT	CCTGGAGAGCCTCCATGAAC
Hspa1a	CAGCGAGGCTGACAAGAAGAA	GGAGATGACCTCCTGGCACT
Idi1	GGTTCAGCTTCTAGCGGAGA	TCGCCTGGGTTACTTAATGG
Klf4	CTGAACAGCAGGGACTGTCA	GTGTGGGTGGCTGTTCTTTT
Lgals3	AGGAGAGGGAATGATGTTGCC	GGTTTGCCACTCTCAAAGGG
Ly6a	TCAGGAGGCAGCAGTTATTGTGGA	TACATTGCAGAGGTCTTCCTGGCA
Mvd	ATGGCCTCAGAAAAGCCTCAG	TGGTCGTTTTTAGCTGGTCCT

Pcnt	GAGGAGAAGTCGGTCTTGTGGA	GCGGTCCTTTTCAGACTGCTTC
Vcam1	CCGGCATATACGAGTGTGAA	GATGCGCAGTAGAGTGCAAG
Xbp1 (spliced)	CTGAGTCCGAATCAGGTGCAG	GTCCATGGGAAGATGTTCTGG
Xbp1 (unspliced)	CAGCACTCAGACTATGTGCA	GTCCATGGGAAGATGTTCTGG

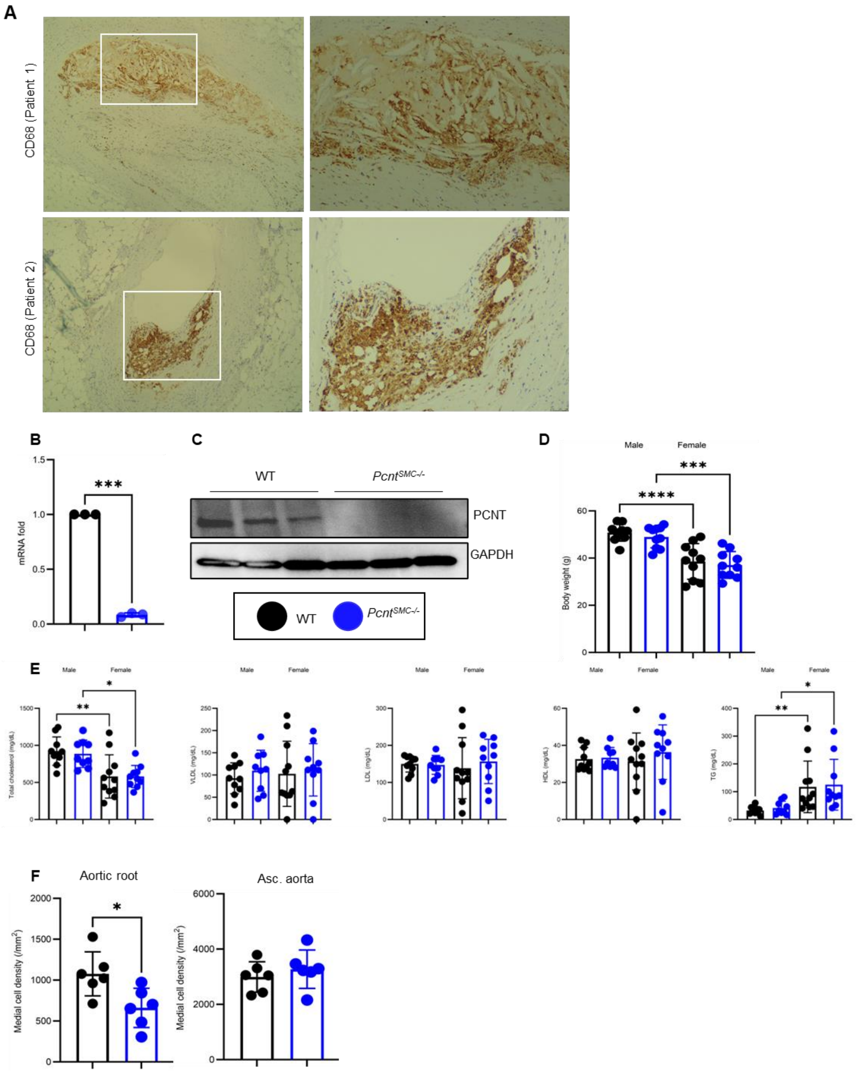

Supplemental Figure 1. Loss of *Pcnt* from SMCs augments atherosclerosis. (A) Coronary artery atherosclerotic lesions of two MOPDII patients show positive staining for CD68 (scale bars: left panels - 250µm, right panels – 100µm). **(B, C)** *Pcnt* mRNA expression (**B**, analyzed by unpaired student's t-test followed by Welch's correction) and immunoblotting confirm successful deletion of PCNT protein from SMCs (**C**)**.** **(D)** Male mice of both genotypes exhibit significantly higher body weights compared to their corresponding females after 12 week HFD (analyzed by 2-way ANOVA followed by Tukey's multiple comparisons test)**.** (**E**) FPLC analysis of serum samples demonstrated no significant difference in total cholesterol, VLDL, LDL, HDL and triglyceride (TG) levels between *Pcnt*$^{SMC-/-}$ and WT mice. Female mice of both genotypes have lower total cholesterol and higher TG compared to their corresponding male mice. N=10 per genotype, per sex. All data were analyzed by 2-way ANOVA followed by Tukey's multiple comparisons test, except for TG, which was analyzed using Kruskal-Wallis test followed by Dunn's multiple comparisons test. (**F**) Aortic roots of *Pcnt*$^{SMC-/-}$ mice showed significantly lower medial cell density compared to WT mice, but no difference was observed in the ascending aortas (unpaired student's t-test followed by Welch's correction). All gene expression data are representative of three independent experiments. Error bars represent standard deviation. *, **, ***, **** - p <0.05, 0.01, 0.001 and 0.0001 respectively.

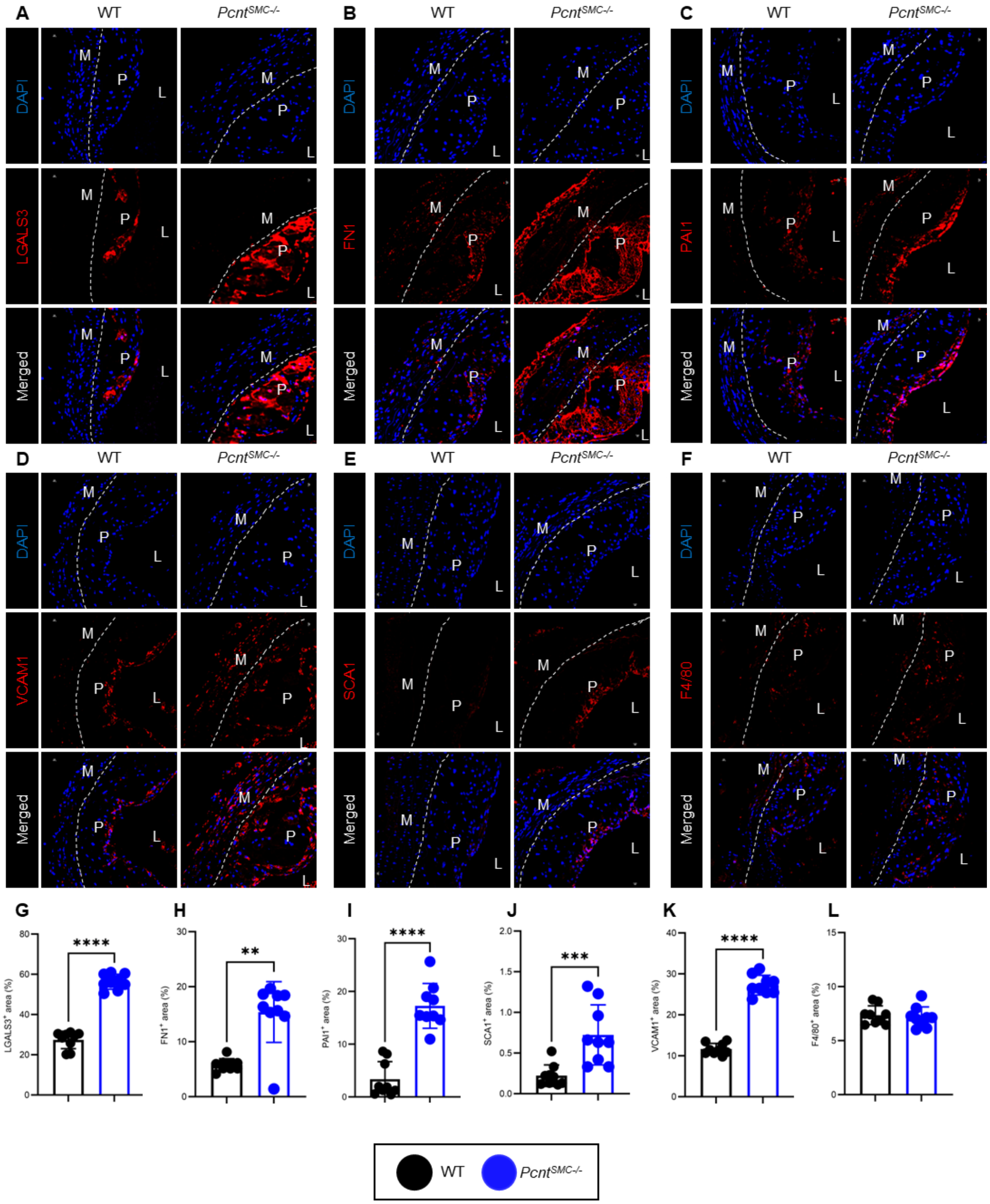

Supplemental Figure 2. *Pcnt*$^{SMC-/-}$ mice show significantly increased expression of SMC modulation markers in ascending aorta lesions. (**A-F**) Immunohistochemical staining of ascending aortic sections against SMC modulation markers LGALS3, FN1, PAI1, VCAM1, SCA1 and the macrophage marker F4/80 (counterstained with DAPI, blue), shows significantly higher staining for all modulation markers except SCA1, and no change in staining for F4/80 in *Pcnt*$^{SMC-/-}$ mice compared to WT mice, as quantified in **G-L.** (N=9, quantification for LGALS3, FN1, PAI1, SCA1, and F4/80 were analyzed by unpaired student's t-test followed by Welch's correction, and that for VCAM1 was analyzed by Mann Whitney U-test). Error bars represent standard deviation. **** - $p < 0.0001$. L, lumen; M, medial layer; and P, plaque. Scale bar – 5μm.

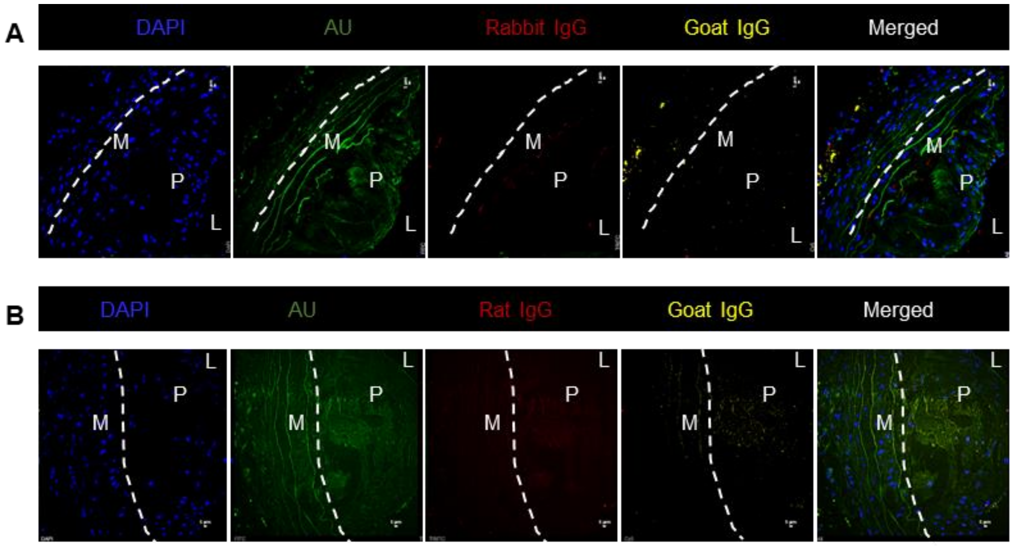

Supplemental Figure 3. Negative controls for immunostaining experiments. (A,B) Aortic sections were stained with normal goat, rabbit or rat IgG to ensure specificity of goat, rabbit and rat antibodies used for immunostaining. Scale bar – 5μm.

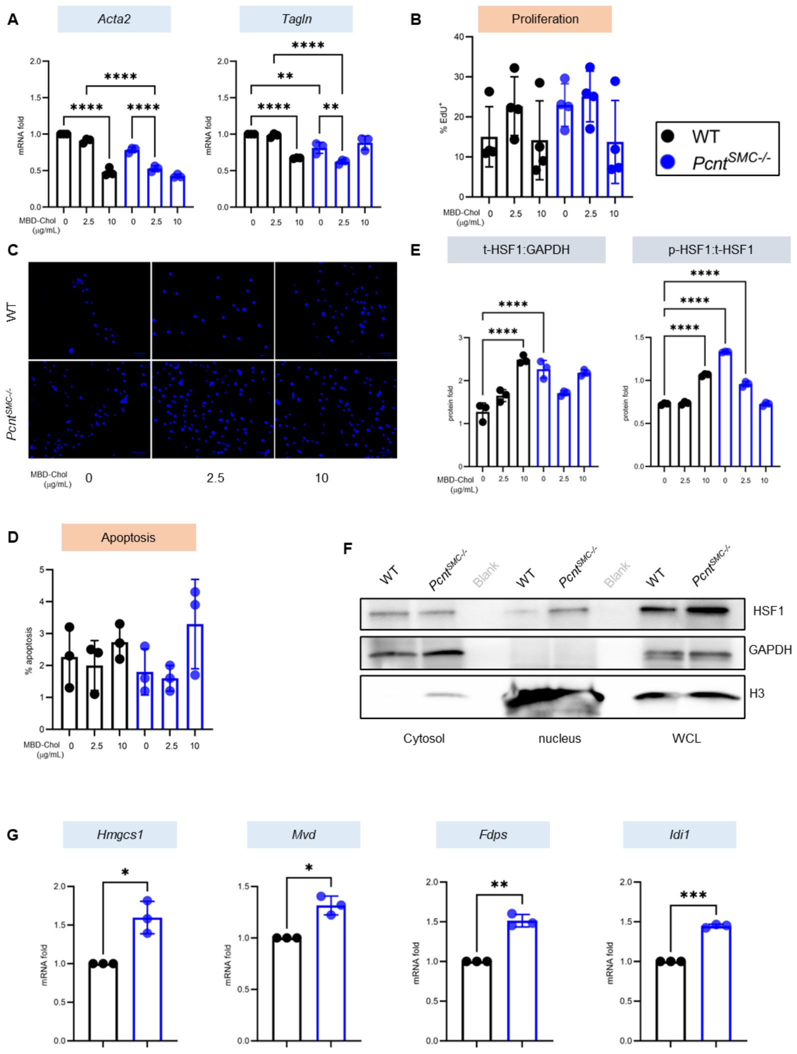

Supplemental Figure 4. Augmented phenotypic modulation of *Pcnt*$^{SMC-/-}$ SMCs is due to increased HSF1 activation driving cholesterol biosynthesis. (A) Expression of contractile makers (*Acta2*, *Tagln*) is reduced at baseline or with exposure to 2.5µg/mL MBD-Chol in *Pcnt*$^{SMC-/-}$ SMCs, compared to with 10µg/mL MBD-Chol in WT SMCs. (**B**) No difference in proliferation, measured by EdU incorporation, is observed between *Pcnt*$^{SMC-/-}$ and WT SMCs. (**C**) *Pcnt*$^{SMC-/-}$ SMCs exhibit higher migration both at baseline and with exposure to MBD-Chol, as demonstrated by DAPI staining of the nuclei of migrated cells (quantified in Main Fig. 4F). (**D**) Flow cytometry analysis of annexin V cells shows no change in apoptosis between genotypes or with cholesterol treatment. (**E**) Quantification of total and phosphorylated HSF1 (p-HSF1) protein levels. Representative blot is shown In Figure 4C. (**F**) Western blots of fractionated lysates show increased nuclear levels of HSF1 in *Pcnt*$^{SMC-/-}$ SMCs. (**G**) Expression of cholesterol biosynthesis pathway genes, *Hmgcs1*, *Mvd*, *Fdps*, *Idi1*, was significantly elevated in *Pcnt*$^{SMC-/-}$ SMCs. Data analyzed by unpaired t-test with Welch's correction. All gene expression data are representative of three independent experiments. Error bars represent standard deviation. All data in A, B and D were analyzed by 2-way ANOVA followed by Tukey's multiple comparisons test, and those in E by unpaired student's t-test followed by Welch's correction. *, **, ***, **** - p <0.05, 0.01, 0.001 and 0.0001 respectively. WCL, whole cell lysates.

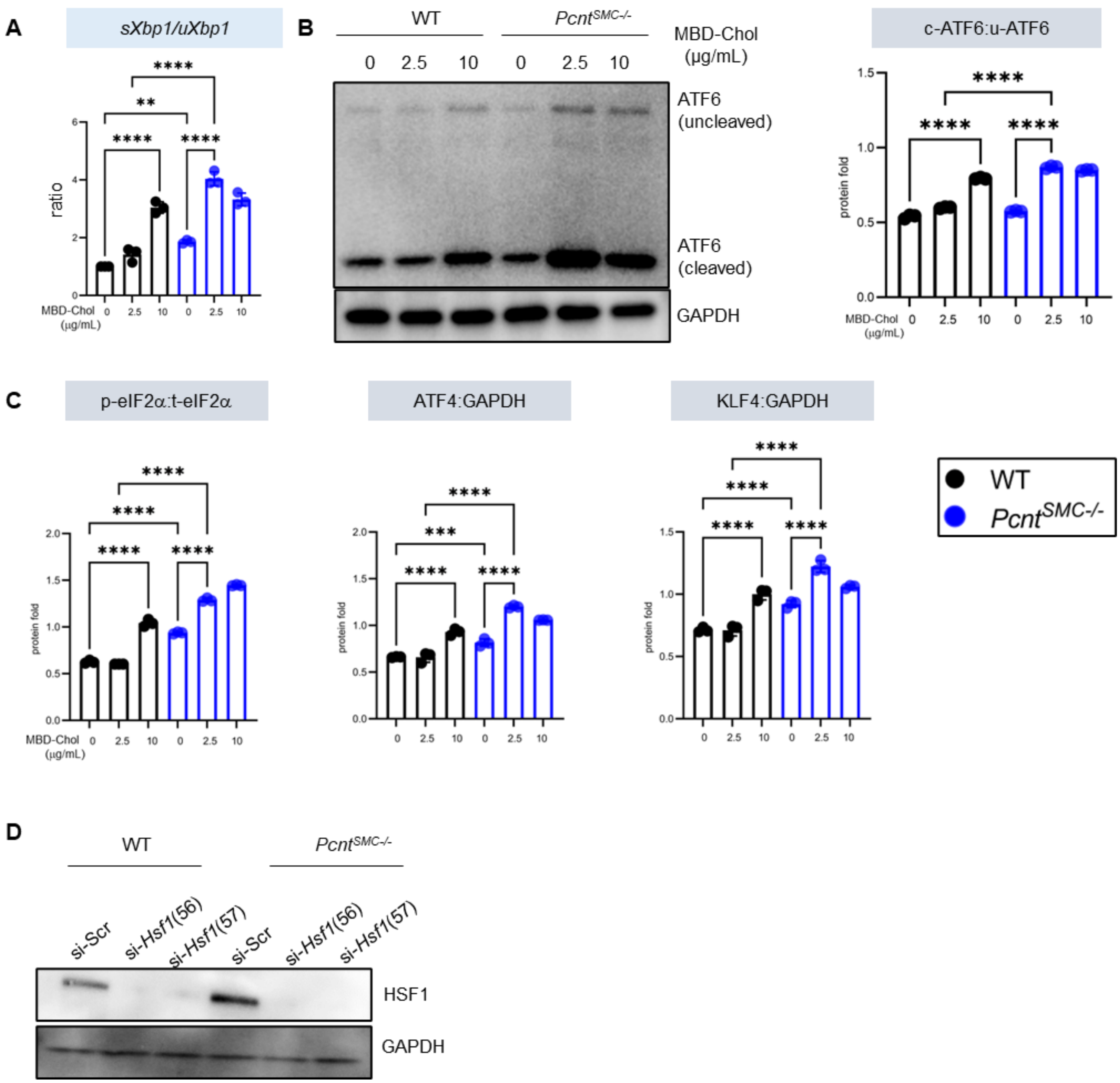

Supplemental Figure 5. ***Pcnt*** **deletion-induced augmented UPR, PERK signaling and SMC phenotypic modulation are HSF1-dependent**. **A,B)** Increased splicing of *Xbp1* (s*Xbp1*/u*Xbp1* ratio) (**A**) and ATF6 cleavage (**B**) demonstrate activation of IRE1α and ATF6 signaling respectively at baseline in *Pcnt*$^{SMC-/-}$ SMCs. (**C**) Quantification of total and phospho-eIF2α, ATF4 and KLF4**.** Confirmation of si-RNA mediated knock down of HSF1. Representative blot is shown in Figure 5B **D)** Western blot confirms knockdown efficiency of two siRNAs directed against *Hsf1*. All data were analyzed by 2-way ANOVA followed by Tukey's multiple comparisons test. All gene expression data are representative of three independent experiments. Error bars represent standard deviation. **, ***, **** - p <0.01, 0.001 and 0.0001 respectively.

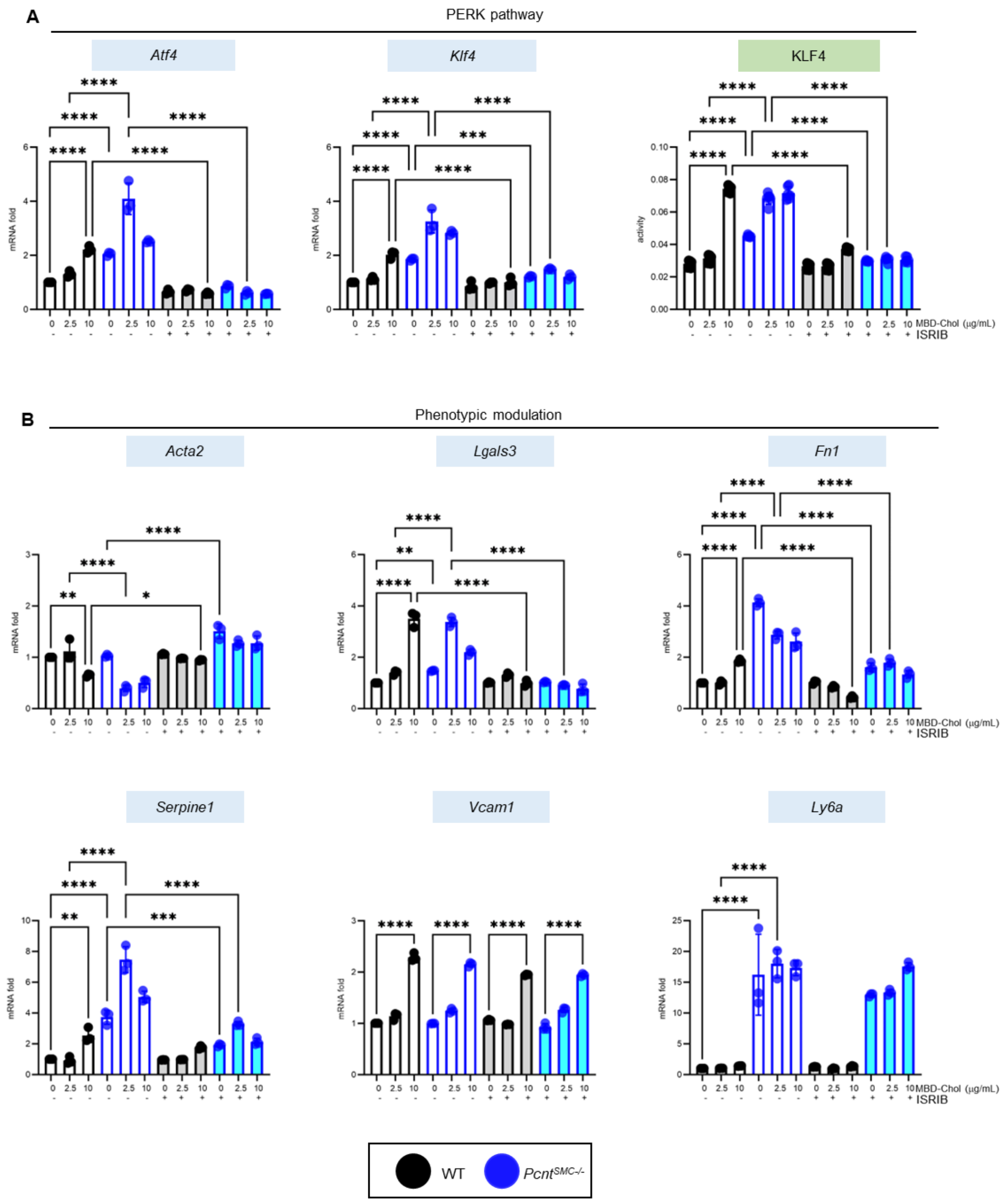

Supplemental Figure 6. Inhibition of PERK signaling reverses augmented phenotypic modulation in *Pcnt*$^{SMC-/-}$ SMCs. (**A**) Treatment with the PERK inhibitor, ISRIB reverses the upregulation of PERK signaling and augmented phenotypic modulation in *Pcnt*$^{SMC-/-}$ SMCs: (*Atf4*, *Klf4* expression and KLF4 activity). (**B**) Treatment with the PERK inhibitor, ISRIB rescues decreased expression of a contractile marker (*Acta2*) and upregulation of all modulation markers except *Vcam1* and *Ly6a*. All data analyzed by two way ANOVA followed by Tukey's multiple comparisons test. All gene expression data are representative of three independent experiments. Error bars represent standard deviation. *, **, ***, **** - p <0.05, 0.01, 0.001 and 0.0001 respectively.

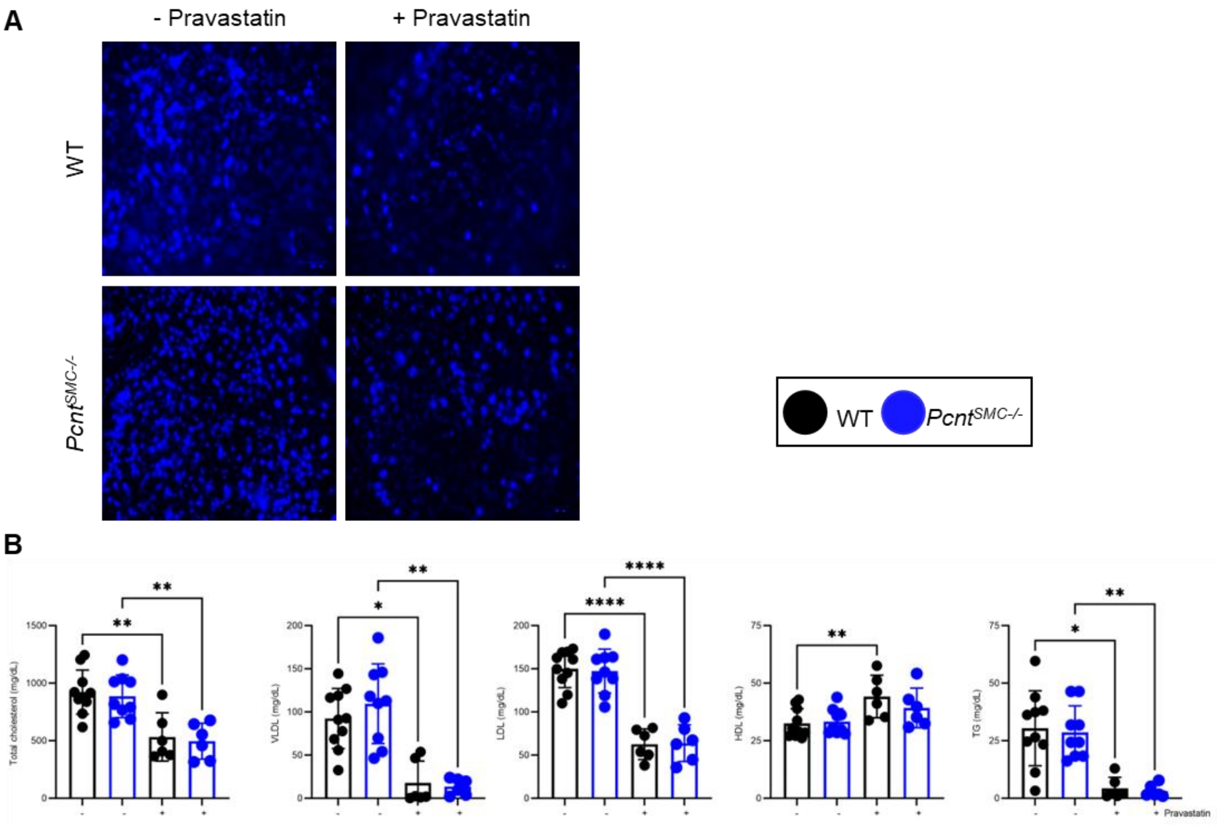

Supplemental Figure 7. Treatment with the HMGCR inhibitor pravastatin reduces migration in *Pcnt*$^{SMC-/-}$ SMCs and serum phospholipids in *Pcnt*$^{SMC-/-}$ mice. (**A**) Pravastatin treatment reduces migration in both *Pcnt*$^{SMC-/-}$ and WT SMCs, quantification show in Figure 6D. (**B**) Lipid profile analysis demonstrated that pravastatin treatment reduced total cholesterol, very low density lipoprotein (VLDL), low density lipoprotein (LDL) and triglyceride (TG) levels in both *Pcnt*$^{SMC-/-}$ and WT mice to a similar extent. N=10 per genotype per treatment. Data was analyzed using Kruskal-Wallis test followed by Dunn's multiple comparisons test for VLDL and TG, and using 2-way ANOVA followed by Tukey's multiple comparisons test for total cholesterol, LDL, and HDL. Error bars represent standard deviation. *, **, **** - p <0.05, 0.01, and 0.0001 respectively.
